# *Bacillus velezensis* LT1: a potential biocontrol agent for southern blight on *Coptis chinensis*

**DOI:** 10.3389/fmicb.2024.1337655

**Published:** 2024-03-04

**Authors:** Tao Tang, Fanfan Wang, Houyun Huang, Jie Guo, Xiaoliang Guo, Yuanyuan Duan, Xiaoyue Wang, Qingfang Wang, Jingmao You

**Affiliations:** ^1^Key Laboratory of Chinese Herbal Medicine Biology and Cultivation, Ministry of Agriculture and Rural Affairs, Institute of Chinese Herbal Medicine, Hubei Academy of Agricultural Science, Enshi, China; ^2^Hubei Engineering Research Center of Good Agricultural Practices (GAP) Production for Chinese Herbal Medicines, Institute of Chinese Herbal Medicines, Hubei Academy of Agricultural Sciences, Enshi, China

**Keywords:** *Bacillus velezensis*, *Sclerotium rolfsii*, *Coptis chinensis*, lipopeptide antimicrobial compounds, biocontrol potential

## Abstract

**Introduction:**

Southern blight, caused by *Sclerotium rolfsii*, poses a serious threat to the cultivation of *Coptis chinensis*, a plant with significant medicinal value. The overreliance on fungicides for controlling this pathogen has led to environmental concerns and resistance issues. There is an urgent need for alternative, sustainable disease management strategies.

**Methods:**

In this study, *Bacillus velezensis* LT1 was isolated from the rhizosphere soil of diseased *C. chinensis* plants. Its biocontrol efficacy against *S. rolfsii* LC1 was evaluated through a confrontation assay. The antimicrobial lipopeptides in the fermentation liquid of *B. velezensis* LT1 were identified using Matrix-Assisted Laser Desorption/Ionization Time-of-Flight Mass Spectrometry (MALDI-TOF-MS). The effects of *B. velezensis* LT1 on the mycelial morphology of *S. rolfsii* LC1 were examined using scanning electron microscopy (SEM) and transmission electron microscopy (TEM).

**Results:**

The confrontation assay indicated that *B. velezensis* LT1 significantly inhibited the growth of *S. rolfsii* LC1, with an inhibition efficiency of 78.41%. MALDI-TOF-MS analysis detected the presence of bacillomycin, surfactin, iturin, and fengycin in the fermentation liquid, all known for their antifungal properties. SEM and TEM observations revealed that the mycelial and cellular structures of *S. rolfsii* LC1 were markedly distorted when exposed to *B. velezensis* LT1.

**Discussion:**

The findings demonstrate that *B. velezensis* LT1 has considerable potential as a biocontrol agent against *S. rolfsii* LC1. The identified lipopeptides likely contribute to the antifungal activity, and the morphological damage to *S. rolfsii* LC1 suggests a mechanism of action. This study underscores the importance of exploring microbial biocontrol agents as a sustainable alternative to chemical fungicides in the management of plant diseases. Further research into the genetic and functional aspects of *B. velezensis* LT1 could provide deeper insights into its biocontrol mechanisms and facilitate its application in agriculture.

## Graphical abstract

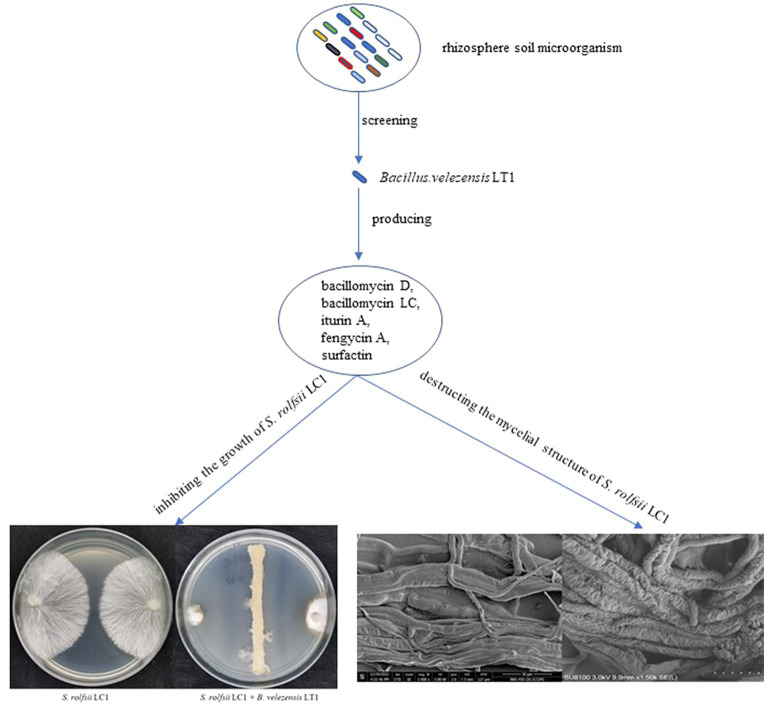

## 1 Introduction

The rhizome of the perennial herb *Coptis chinensis*, also known as goldthread in Chinese medicine, has been commonly used alone or in combination with other herbs in many traditional Chinese medicine formulations (Wang et al., 2019). Due to its extensive antibacterial, antiviral, and antioxidant effects, it is often used to treat inflammatory diseases, diabetes, cardiovascular diseases, cancer, and neurodegenerative diseases (Zhen et al., 2011; Chou et al., 2017). Southern blight caused by *Sclerotium rolfsii* is one of the most serious diseases affecting *C. chinensis*, with a field incidence of approximately 10–20% and severe cases of up to 80%. Furthermore, southern blight infections have a considerable impact on the yield and quality of the herb and can even result in no harvest. Currently, the control of southern blight on *C. chinensis* mainly relies on the use of chemical fungicides. Only a few registered drugs, such as thifuramide and fluoxafen, are available for the prevention of southern blight (China Pesticide Information Network, http://www.chinapesticide.gov.cn). Excessive use of a single fungicide can lead to the rapid development of drug resistance, resulting in a decrease in the prevention and control effect (Mikaberidze et al., 2017). Furthermore, the excessive use of chemical agents can have an immeasurable impact on the quality of *C. chinensis*. Therefore, it is urgent to find a new green and efficient control method for southern blight on *C. chinensis*.

As public demand for eco-friendly and healthy food options surges, coupled with heightened environmental consciousness, biological control strategies are garnering increasing interest for their sustainable and eco-friendly attributes. Among a variety of pathogens, microbial antagonists have emerged as a focal point among a diverse array of pathogens due to their recognized safety, effectiveness, and environmentally benign characteristics (Li et al., 2022). These antagonists are emerging as trustworthy substitutes for traditional chemical agents. Over the past several decades, research has highlighted the pivotal role that microorganisms play in the plant rhizosphere soil in combatting plant pathogens, marking a critical advancement in agricultural sciences (Cakmakci et al., 2006; Mehta et al., 2014; Manuel et al., 2023). Plant growth-promoting rhizobacteria (PGPR) are beneficial bacteria found in the plant rhizosphere. They are capable of colonizing the root surface and interior, enhancing plant growth and suppressing disease occurrence (Oleńska et al., 2020). PGPR facilitate plant growth through a variety of mechanisms, such as the production of iron carriers, increased phosphorolysis, improved nutrient absorption, and the secretion of plant hormones (Backer et al., 2018). Moreover, many PGPRs can prevent the recurrence of diseases by inducing systemic resistance (ISR), engaging in interspecific competition, and emitting volatile organic compounds (VOCs) (Li et al., 2020). Soil-beneficial microorganisms are important sources of PGPRs, such as *Bacillus*, *Streptomyces*, *Pseudomonas*, *Burkholderia*, and *Trichoderma*, and all of them are isolated from rhizosphere soil, being widely used in disease control and soil improvement in agricultural production (Le et al., 2022; Liu et al., 2022; Venturi, 2022; Xue et al., 2022; Baard et al., 2023). Screening beneficial microorganisms from soil to combat pathogens is also a crucial strategy in plant disease management.

*Bacillus velezensis* was first identified by Ruiz-García et al. in 2005 and has been shown to produce multiple compounds that can antagonize plant pathogens (Ruiz-García et al., 2005). *B. velezensis* has been found to produce numerous active substances, such as surfactins, fengycins, bacillomycin-D, bacillaene, macrolactin, difficidin, bacillibactin, and bacilysin, which are involved in inhibiting the growth of plant pathogens (Gao et al., 2017; Rabbee et al., 2019). To date, several strains of *B. velezensis* have been reported to have the potential to be used for controlling plant diseases and promoting plant growth (Zhang et al., 2019; Kim et al., 2022; Sun et al., 2022). In this study, we aimed to identify rhizosphere growth-promoting bacteria that could inhibit southern blight of *C. chinensis* in rhizosphere soil. To achieve green prevention and control of southern blight on *C. chinensis*, we conducted the following work to screen for satisfactory biocontrol bacteria: (1) isolated and identified the bacterial community in the rhizosphere soil of *C. chinensis* and verified its inhibitory effect on *S. rolfsii*, (2) clarified the biological characteristics and control effect of *B. velezensis* LT1 against southern blight, and (3) clarified the types of lipopeptide antibiotics produced by *B. velezensis* LT1.

## 2 Materials and methods

### 2.1 Fungal and bacterial strains

All fungal and bacterial strains used in this study were isolated and preserved by the Institute of Chinese Herbal Medicines, Hubei Academy of Agricultural Sciences. The main strains, *B. velezensis* LT1 and *S. rolfsii* LC1, were stored in the China Center for Type Culture Collection Center with the numbers of CCTCC M 2020023 and CCTCC M 20231389, respectively.

### 2.2 Sample collection

Rhizosphere soil samples were collected from the *C. chinensis* GAP planting base (108°35′E, 30°22′N) located in Lichuan City, Enshi Prefecture, Hubei Province, in October 2019. The planting base had a history of perennial southern blight occurrence. The rhizosphere soil, which is located approximately 5–10 cm below the ground part of the *C. chinensis* plant, was collected using a JC-802 root soil sampler (Qingdao Juchuang Times Environmental Protection Technology Co., LTD, Qingdao, China). The rhizome samples obtained from five locations in the rhizosphere soil were uniformly mixed to form one sample.

### 2.3 Isolation and identification of bacterial strains

Bacteria in the rhizosphere soil of *C. chinensis* were isolated using the dilution coating method, as described by Li et al. (2023). The soil samples were prepared into suspensions with concentrations of approximately 10^−1^–10^−6^, and 50 μL of the soil dilutions was uniformly coated on the surface of NA medium, R2A medium, and brain-heart infusion liquid medium. The NA medium contained 10 g of tryptone, 3 g of beef powder, 5 g of NaCl, and 15 g of agar per liter. The R2A medium contained 0.5 g of yeast extract powder, 0.5 g tryptone, 0.5 g of casein hydrolysate, 0.5 g of glucose, 0.5 g soluble starch, 0.3 g of KH_2_PO_4_, 0.024 g of MgSO_4_, 0.3 g of CH_3_COCOONa, and 15 g of agar per liter. The brain-heart infusion liquid medium contained 5 g of tryptone, g of glucose, 5 g of NaCl, 2.5 g of Na_2_HPO_4_, 4 g of beef brain infusion, 16 g of casein peptone, and 15 g of agar per liter. The Petri dishes were then sealed and incubated at a constant temperature of 28°C. Colonies with distinct differences in morphology, size, and color were selected for purification. The colonies were purified three to four times using the scribing plate method until single colonies were observed. Single colonies were transferred to a bead-side culture medium test tube and stored in the refrigerator at 4°C for future use. Frozen stocks of all the purified colonies were prepared using 25% glycerol and stored at −80°C.

Bacterial genomic DNA was extracted using a Soil FastDNA SPIN Kit (MP Biomedicals (Shanghai) Co., Ltd., Shanghai, China). After assessing the quality of the genomic DNA extraction using a microspectrophotometer (NanoDrop One C, Thermo Fisher Scientific, Madison, United States), 16S rDNA was amplified using the primers 24F (5′-GTTTGATCCTGGCTCAG-3′) and 1494R (5′-ACGGCTACCTTGTTACGACTT-3′). The PCR products were purified and recovered by Wuhan Tsingke Biology Co., Ltd. for sequencing. The sequencing results were compared with the BLAST® sequence analysis tool at the US National Center for Biotechnology Information (NCBI) for preliminary identification of the isolated strains.

### 2.4 Screening of antagonistic strains

The plate confrontation method (Yue et al., 2023) was used to screen for antagonistic strains against *S. rolfsii* in *C. chinensis*. The activated *S. rolfsii* LC1 was inoculated with a mycelial plug using a hole punch of 5 mm diameter and placed in the center of the PDA medium with the mycelial side facing down. The isolated and purified bacteria were inoculated 2.5 cm away from the mycelial plug and cultured at 28°C for 4–7 days. The control group was not inoculated with the isolated strains, and each experiment was repeated three times. The colony diameter was measured using the crisscross method, and the inhibition rate was calculated using the following formula:


$$\begin{array}{l} \mathrm{Inhibition}\;\mathrm{rate}\;\left(\%\right)\\ =\frac{\left(\mathrm{control}\;\mathrm{group}\;\mathrm{colony}\;\mathrm{diameter}-\mathrm{treatment}\;\mathrm{group}\;\mathrm{colony}\;\mathrm{diameter}\right)}{\mathrm{control}\;\mathrm{group}\;\mathrm{colony}\;\mathrm{diameter}}\times 100 \end{array}$$

### 2.5 Morphological, biological characteristics, and sequence analysis of antagonistic bacteria LT1

The LT1 strain exhibited the strongest inhibitory effect on *S. rolfsii* LC1. Therefore, only the LT1 strain was subjected to morphological and molecular analyses. Morphological characteristics of the LT1 strain were observed using microscopy LSM 900 (Carl Zeiss Co. Ltd., Chengdu, China) and scanning electron microscopy (HITACHI Regulus 8,100) (Hitachi Production Co., LTD., Tokyo, Japan). Subsequently, the LT1 strain underwent Gram staining using the Gram Stain Kit HB8278 (Haibo Biotechnology Co. LTD., Qingdao, China). The *gyrA* gene was amplified using the primers gyrA-F (5’-CAGTCAGGAAATGCGTACGTCCTT-3′) and gyrA-R (5’-CAAGGTAATGCTCCAGGCATTGCT-3′). The PCR products were subjected to 1% (w/v) agarose gel electrophoresis, purified, and sequenced by Wuhan Aoke Biotechnology Co., Ltd. The sequencing results were compared using the BLAST® sequence analysis tool in NCBI^1^ and nucleic acid homology comparison with the data in GenBank. When constructing a phylogenetic tree using *16S rDNA* and *gyrA* genes, bacteria with high homology to *Bacillus* were selected as ingroups and other bacteria were selected as outgroups. Clustal X was used to compare the sequences, and MEGA5.0 software (Mega Technology Co. LTD., Suzhou, China) was used to analyze the phylogeny of all bacteria. The two genes were combined to construct a phylogenetic tree, and the bootstrap value was tested 1,000 times. Finally, the optimum growth temperature and pH for the LT1 strain were determined.

### 2.6 Antagonistic activity of LT1 strain fermentation liquor

To obtain the fermentation liquor, 1 mL of the LT1 strain was inoculated into 1,000 mL of liquid LB and incubated for 48 h at 180 r/min at 28°C. The fermentation solution was centrifuged at 3000 r/min for 10 min at 4°C, and the upper fermentation solution was filtered using a bacterial filter (0.22 μm). The inhibition rate of the resulting fermentation liquor on the mycelial growth of *S. rolfsii* LC1 was determined using a solid plate method. The LT1 fermentation liquor was prepared in 20 mL PDA plates at proportions of 2, 6, and 10% (v/v). PDA plates without the fermentation filtrate were used as controls. The mycelial plug of activated *S. rolfsii* LC1 (φ = 5 mm) was inoculated in the center of the PDA plate and cultured at 28°C for 3 days. The diameter of each colony was measured using the criss-cross method with three replicates per treatment. The mycelial growth inhibition rate was calculated using the following formula:


$$\begin{array}{l} \mathrm{Inhibition}\;\mathrm{rate}\;\left(\%\right)\\ =\frac{\left(\mathrm{control}\;\mathrm{group}\;\mathrm{colony}\;\mathrm{diameter}-\mathrm{treatment}\;\mathrm{group}\;\mathrm{colony}\;\mathrm{diameter}\right)}{\mathrm{control}\;\mathrm{group}\;\mathrm{colony}\;\mathrm{diameter}}\times 100 \end{array}$$

Since the 10% fermentation liquor exhibited the best inhibitory effect against *S. rolfsii* LC1, it was selected to assess the effect of LT1 strain against other pathogens such as *Rhizoctonia solani* ZJSQK1 of *Panax japonicus*, *Sclerotinia sclerotiorum* BXHP1 of *Pinellia ternata*, *Fusarium acuminatum* MD1 of *Ophiopogon japonicus*, *Fusarium oxysporum* QK-4 of tobacco, *Colletotrichum micotianae* YCTJ1 of tobacco, *Alternaria alternata* YCCX1 of tobacco, and *Botrytis cinerea* ES8 of *Paris polyphylla*. All the aforementioned pathogens were obtained from the Institute of Chinese Herbal Medicines of the Hubei Academy of Agricultural Sciences and preserved.

### 2.7 Inhibitory effect of LT1 strain against southern blight on potted *Coptis chinensis*

First, the fermentation liquid and filtered liquid of the LT1 strain were directly sprayed onto potted *C. chinensis*, and then, healthy leaves were selected to inoculate the activated *S. rolfsii* LC1. Each Potted *C. chinensis* was inoculated with three leaves. Potted *C. chinensis* inoculated with LC1 was then sealed in a plastic bucket to maintain moisture, and the incidence of southern blight was observed. Untreated *C. chinensis* were inoculated with *S. rolfsii* LC1 as a control group, which were treated with tifuramide as a positive control group, and each experiment was repeated three times.

### 2.8 Effects of different extractants on inhibition activity of strain LT1 fermentation filtrate

From 150 mL of LT1 strain’s fermentation broth, 20 mL of acetic acid was incorporated to attain a pH of 3.0. The fermentation filtrate was then sequentially extracted with equal volumes (1:1 v/v) of ethyl acetate, dichloromethane, methanol, and acetone. The extracts were clarified by passing them through a 0.22 μm filter to remove any bacterial residues. Subsequently, these filtered extracts were incorporated into a PDA medium at a 1:10 (v/v) ratio to prepare agar plates. An actively growing mycelial plug of *S. rolfsii* LC1 (φ = 5 mm) was centrally inoculated onto each PDA plate. The plates were then incubated at 28°C for 3 days. As a control, an equal volume of each solvent was added to separate PDA plates. The experiment was conducted in triplicate for statistical robustness. The diameter of each treated colony was determined by the cross-cross method. The mycelial growth inhibition rate was calculated using the following formula:


$$\begin{array}{l} \mathrm{Inhibition}\;\mathrm{rate}\;\left(\%\right)\\ =\frac{\left(\mathrm{control}\;\mathrm{group}\;\mathrm{colony}\;\mathrm{diameter}-\mathrm{treatment}\;\mathrm{group}\;\mathrm{colony}\;\mathrm{diameter}\right)}{\mathrm{control}\;\mathrm{group}\;\mathrm{colony}\;\mathrm{diameter}}\times 100 \end{array}$$

### 2.9 Identification of Lipopeptide antimicrobial compounds produced by the LT1 strain

The fermentation filtrate was extracted with a 1:1 (v:v) acetone solution. In total, 1 μL of the acetone extract was placed on a target containing the same amount of mother liquor (CHCA) in the mass spectrometer and air-dried naturally. Matrix-assisted laser desorption/ionization time-of-flight mass spectrometry (MALDI-TOF-MS) (Waters Corporation, Milford, MA, United States) was used for mass spectrometry analysis by a Crylas FTS355-Q laser (CryLaS, Berlin, Germany) source (355 nm) as the ion analytical ionization source, with a frequency of 1,000 HZ, laser energy of 250, and sample test range of 0–2000 *m/z*.

### 2.10 Effect of LT1 strain on mycelial morphology of *Sclerotium rolfsii* LC1

The fermentation filtrate of LT1 was mixed with PDA medium at a ratio of 1:9 (v/v) to prepare a plate. An activated *S. rolfsii* LC1 mycelial plug (φ = 5 mm) was inoculated into the center of the PDA plate and cultured at 28°C for 3 days. The mycelium was excised and rapidly submerged into glutaraldehyde solution for electron microscopy fixation for 2 hours, then washed three times with 0.1M phosphate buffer (PB) at pH 7.4. The mycelial morphology was observed using a scanning electron microscope (HITACHI Regulus 8,100) (Hitachi Production Co., LTD., Tokyo, Japan) and a transmission electron microscope (HITACHI HT7800) (Hitachi Production Co., LTD., Tokyo, Japan).

### 2.11 Statistical analysis

The data were analyzed using analysis of variance and Duncan’s multiple comparison tests with SPSS 22.0 (International Business Machines Corporation, New York, United States). The mean ± standard error (SEM) was used to express the data. Significance levels were indicated as ^*^ *p <* 0.05 and ^**^ *p <* 0.01 for two levels of significant differences. GraphPad Prism software (version 6.0, GraphPad Software, Calif., United States) was used for data visualization.

## 3 Results

### 3.1 *Bacillus* sp. LT1 isolated from the rhizosphere soil of *Coptis chinensis* significantly inhibited the growth of *Sclerotium rolfsii* LC1

**Figure 1:**
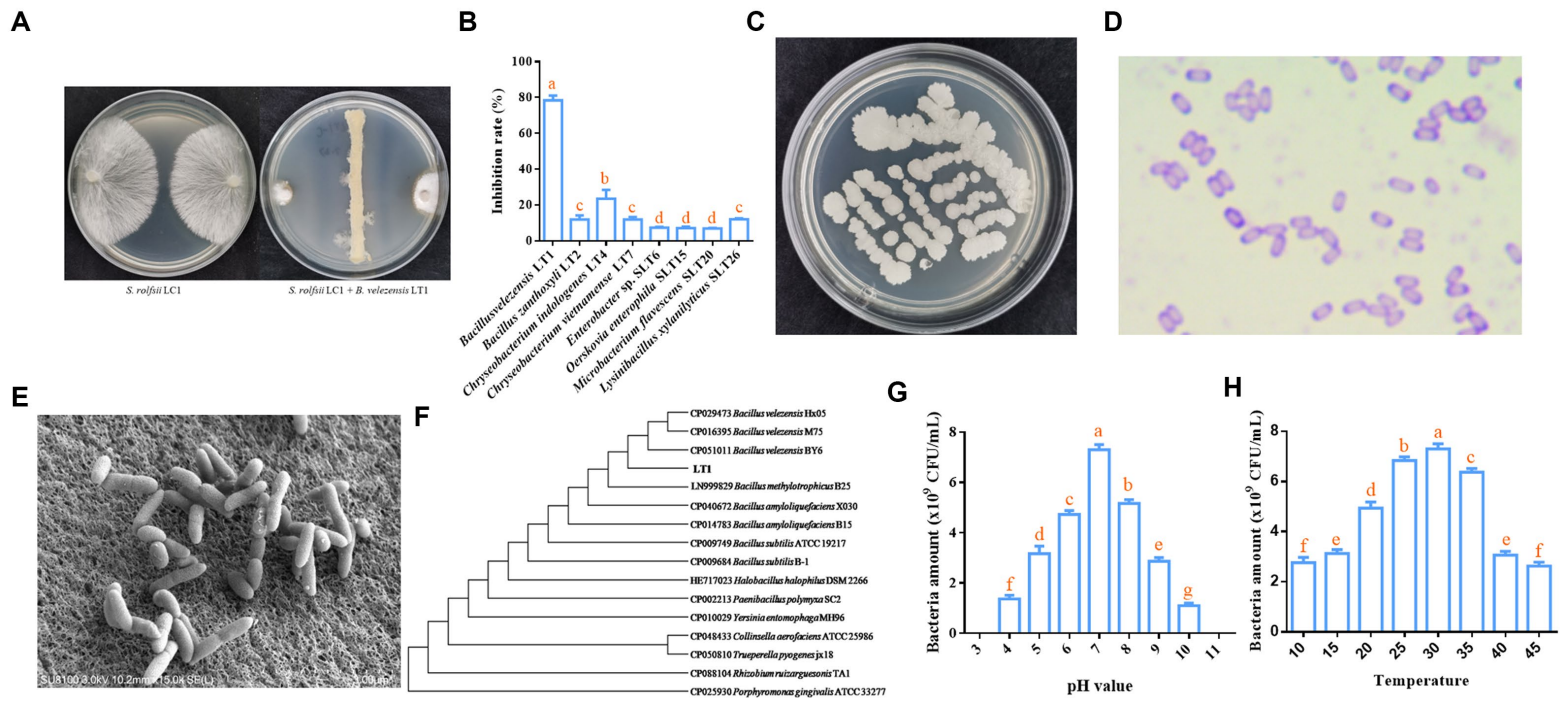
Biological and molecular biological characteristics of LT1 strain. **(A)** Morphology of the LT1 strain inhibiting the growth of *S. rolfsii* LC1; **(B)** Inhibitory effect of rhizosphere soil bacteria on *S. rolfsii* LC1 of *C. chinensis*; **(C)** Colony morphology of the LT1 strain on NA medium; **(D)** Morphology of Gram-stained LT1 strain; **(E)** Morphology of the LT1 strain observed under a scanning electron microscope; **(F)** Phylogenetic tree of LT1 based on 16S rDNA and *gyrA* genes; **(G)** Effect of *pH* on the growth of the LT1 strain; **(H)** Effect of temperature on the growth of the LT1 strain. Error bars represent mean ± standard deviation. One-way analysis of variance was performed using GraphPad Prism 6.0 software. Different letters represent significant differences between different treatment groups (*p* < 0.05).

A total of 46 bacterial strains with different morphologies were obtained by coating the diluted soil suspension onto R2A, BHI, and NA media to obtain single colonies. The 46 strains were preliminarily identified by *16S rDNA* that belong to 4 phyla, 9 classes, 10 orders, 17 families, 24 genera, and 32 species (Supplementary Table S1). The sequencing results of *16S rDNA* for all strains are shown in Supplementary Data S1. The inhibitory effect of LT1 strain on the mycelial growth of *S. rolfsii* LC1 was measured using the face-off culture method. Many strains, including LT1, LT2, LT4, and SLT26, could significantly inhibit the growth of *S. rolfsii* LC1. Among these, the LT1 strain showed the best inhibitory effect (78.41%) (Figures 1A,B).

### 3.2 LT1 Strain was identified as *Bacillus velezensis* LT1

Morphological and molecular biology methods were employed to identify the specific species of the LT1 strain with the best antagonistic effect. The single colony of LT1 on the NA medium was round, rough, and white (Figure 1C). The purple Gram staining indicated that the LT1 strain was a gram-positive bacterium (Figure 1D). Scanning electron microscopy showed that the bacteria were rod-shaped with obtuse ends and smooth cell walls, ranging in size from 0.3 μm x 0.39 μm to 0.75 μm x 1.53 μm (Figure 1E). The 16S rDNA sequence of the LT1 strain with gene number OL677384 was found to be 99.8% similar to that of *B. velezensis* Blz02 (number: MN961683) from NCBI. The *gyrA* sequence of the LT1 strain with gene number OL692738 was 100% similar to that of *B. velezensis* Blz02 (number: MT395302) from NCBI. Based on the 16S rDNA sequence and *gyrA* sequence, a joint evolutionary tree was constructed, and the LT1 strain was clustered with the *B. velezensis* BY6, M75, and Hx05 strains. It could be effectively distinguished from other bacteria, such as *B. amyloliquefaciens* and *B. subtilis* (Figure 1F). Based on the morphological characteristics and molecular sequences, the LT1 strain was identified as *B. velezensis* LT1. The optimum pH for the growth of *B. velezensis* LT1 was 7, and it could not grow when the pH was lower than 3 or greater than 11 (Figure 1G). The optimum growth temperature was between 25 and 30°C, and *B. velezensis* LT1 could grow at temperatures ranging from 10 to 45°C (Figure 1H).

### 3.3 Effectiveness of *Bacillus velezensis* LT1 fermentation liquor in controlling southern blight of *Coptis chinensis*

**Figure 2:**
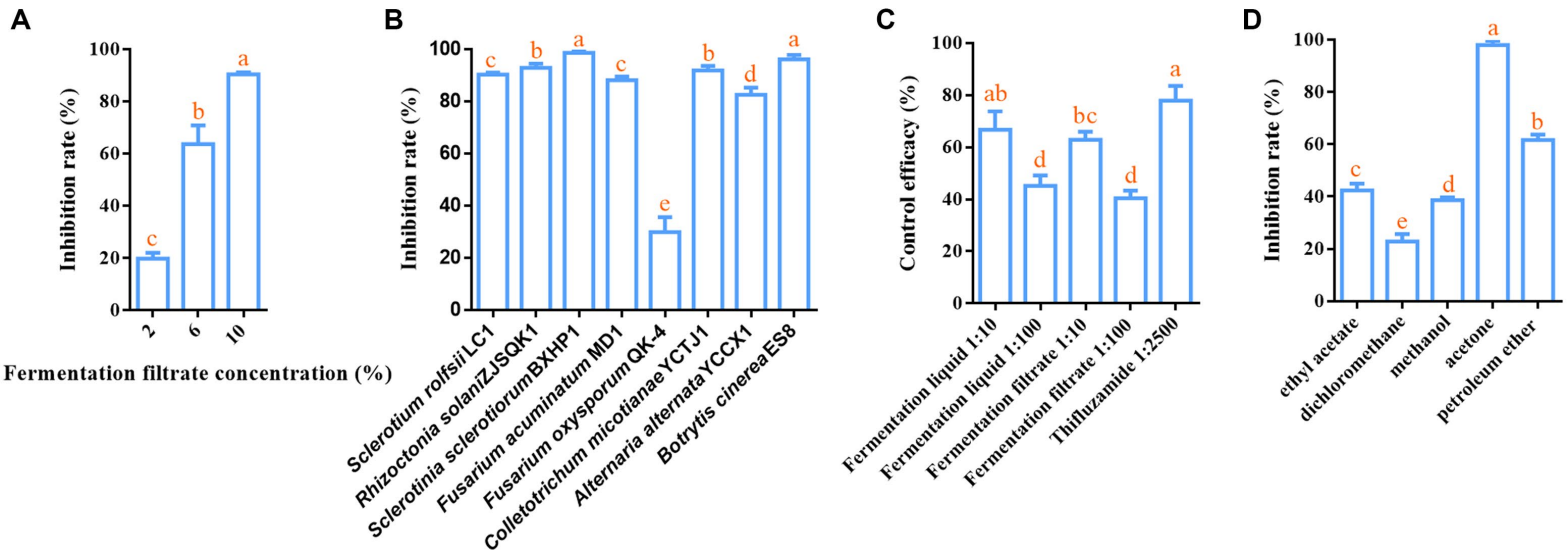
Inhibition effect of *B. velezensis* LT1 fermentation liquid on *S. rolfsii* LC1. **(A)** Inhibitory effect of fermentation liquid at different concentrations in PDA medium on *S. rolfsii* LC1; **(B)** Inhibitory effect of 10% fermentation liquid of *B. velezensis* LT1 on other pathogens; **(C)** Control efficacy of different concentrations of fermentation liquid and fermentation filtrate against southern blight in potted *C. chinensis*; **(D)** Inhibition effect of fermentation liquid of *B. velezensis* LT1 with different solvents extracted from *S. rolfsii* LC1. Error bars represent mean ± standard deviation. One-way analysis of variance was performed using GraphPad Prism 6.0 software. Different letters represent significant differences between different treatment groups (*p* < 0.05).

To assess the biocontrol potential of *B. velezensis* LT1 against southern blight of *C. chinensis*, we measured the inhibitory effect of the fermentation liquor without bacterial cells of *B. velezensis* LT1 on *S. rolfsii* LC1 *in vitro* and its control effect on potted *C. chinensis*. Three concentrations of *B. velezensis* LT1 fermentation filtrate showed inhibitory effects on the growth of *S. rolfsii*, with the inhibitory effects significantly varying among the concentrations. The inhibitory effect increased gradually as the fermentation filtrate concentration increased, reaching 90.43% when the fermentation filtrate concentration reached 10% (Figure 2A). We used a 10% fermentation filtrate to measure the inhibitory effects of *B. velezensis* LT1 on other pathogens. The results showed that *B. velezensis* LT1 had strong inhibitory effects on *R. solani* ZJSQK1 of *P. japonicus*, *S. sclerotiorum* BXHP1of *P. ternata*, *F. acuminatum* MD1 of *O. japonicus*, *C. micotianae* YCTJ1 of tobacco, *A. alternata* YCCX1 of tobacco, and *B. cinerea* ES8 of *P. polyphylla* (Figure 2B). These results demonstrate the high potential of *B. velezensis* LT1 for plant disease control. To further evaluate the control efficacy of *B. velezensis* LT1 against the southern blight of *C. chinensis*, we conducted pot experiments using different concentrations of fermentation liquid and filtrate. The control efficacy of 10% fermentation liquid and fermentation filtrate of *B. velezensis* LT1 against southern blight on potted *C. chinensis* reached 65%, whereas that of 1% fermentation liquid and fermentation filtrate of *B. velezensis* LT1 was approximately 40% (Figure 2C). The control efficacy of the 10% fermentation liquid was similar to that of the chemical fungicide tifuramide (Figure 2C). Notably, the same concentrations of fermentation liquid and filtrate without bacterial cells of *B. velezensis* LT1 showed equal control efficacy.

### 3.4 *Bacillus velezensis* LT1 Produces a variety of lipopeptide antimicrobial compounds to inhibit the growth of *Sclerotium rolfsii* LC1

The fermentation liquid without bacterial cells of *B. velezensis* LT1 was found to significantly inhibit the growth of *S. rolfsii* LC1. This led us to suspect that *B. velezensis* LT1 may have produced substances in the fermentation liquid that inhibit fungal growth. To identify the specific components of these substances, *B. velezensis* LT1 fermentation liquid was extracted using different solvents. Among the five extractants tested, the fermentation liquid extracted with acetone showed the best inhibitory effect with a mycelial inhibition rate of *S. rolfsii* LC1 of 99%, followed by petroleum ether extraction with an inhibition rate of 61.28%. The inhibition rates of the extraction products using the other three extractants were lower than 45% (Figure 2D). Therefore, the acetone extract of *B. velezensis* LT1 fermentation liquid was subjected to MALDI-TOF-MS (*m/z* 0–2000) analysis, and the resulting lipopeptide secondary metabolites (*m/z* 0–2000) are shown in Supplementary Data S2. *B. velezensis* LT1 was found to produce multiple antimicrobial compounds, including bacillomycin D, bacillomycin LC, iturin A, fengycin A, and surfactin (Table 1). Many of these antimicrobial compounds have been reported to inhibit the growth of fungal pathogens. These results suggest that the production of rich lipopeptide antimicrobial compounds by *B. velezensis* LT1 may be the primary mechanism by which it inhibits *S. rolfsii* LC1.

**Table 1:** Lipopeptide antibiotics substances produced by *B. velezensis* LT1.

Antibiotic name	m/*z*	Ion type	Compound
Bacillomycin	1053.5,1053.6,1069.5	[M + H]^+^, [M + Na]^+^,[M + K]^+^	Bacillomycin D (C_11_)
	1066.7,1067.5,1069.4,1069.6,1083.5	[M + Na]^+^, [M + K]^+^	Bacillomycin D (C_12_)
	1081.5,1081.7,1083.5,1097.5	[M + Na]^+^, [M + K]^+^	Bacillomycin D (C_13_)
	1095.5,1099.7,1111.5,1111.6,1111.7	[M + Na]^+^, [M + K]^+^	Bacillomycin D (C_14_)
	1109.5	[M + Na]^+^	Bacillomycin D (C_15_)
	1063.6	[M + H]^+^	Bacillomycin LC (C_14_)
	1099.6	[M + Na]^+^	Bacillomycin LC (C_15_)
Iturin	1065.5,1081.5	[M + Na]^+^,[M + K]^+^	Itutin A (C_14_)
	1079.5,1095.5	[M + Na]^+^,[M + K]^+^	Itutin A (C_15_)
	1093.5,1109.5	[M + Na]^+^,[M + K]^+^	Itutin A (C_16_)
	1066.7,1082.6,1082.7	[M + Na]^+^,[M + K]^+^	Itutin C (C_11_)
	1080.7,1096.6	[M + Na]^+^,[M + K]^+^	Itutin C (C_12_)
	1094.6,1094.7	[M + Na]^+^	Itutin C (C_13_)
	1136.6	[M + Na]^+^	Itutin C (C_16_)
	1150.6	[M + Na]^+^	Itutin C (C_17_)
	1098.6,1098.7,1098.7, 1120.6	[M + H]^+^, [M + Na]^+^	Itutin C (C_15_)
	1112.6,1134.6	[M + H]^+^, [M + Na]+	Itutin C (C_16_)
	1435.0,1435.8,1437.7,1457.8	[M + K]^+^, [M + H]^+^, [M + Na]^+^	Fengycin A (C_14_)
Fengycin	1449.8,1487.7,1471.7,1471.8	[M + K]^+^, [M + H]^+^, [M + Na]^+^	Fengycin A (C_15_)
	1463.8,1,464,1485.7,1485.8,1450.0,1501.7	[M + K]^+^, [M + H]^+^, [M + Na]^+^	Fengycin A (C_16_)
	1477.8,1477.9,1478.0,1499.8,1515.6	[M + K]^+^, [M + H]^+^, [M + Na]^+^	Fengycin A (C_17_)
	1491.9,1492.0,1513.8,1529.8	[M + K]^+^, [M + H]^+^, [M + Na]^+^	Fengycin A (C_18_)
	1505.8,1,506,1527.8,1543.8	[M + K]^+^, [M + H]^+^, [M + Na]^+^	Fengycin A (C_19_)
	1519.7,1557.8	[M + K]^+^, [M + H]^+^	Fengycin A (C_20_)
	1533.8,1571.8	[M + K]^+^, [M + H]^+^, [M + Na]^+^	Fengycin A (C_21_)
	1547.8	[M + H]^+^	Fengycin A (C_22_)
Surfactin	1030.7,1046.6	[M + K]^+^, [M + Na]^+^	Surfactin (C_11_)
	1060.6	[M + K]^+^, [M + Na]^+^	Surfactin (C_12_)
	1058.7,1058.8,1059.7,1072.6	[M + K]^+^, [M + Na]^+^	Surfactin (C_13_)
	1088.6,1088.7	[M + K]^+^	Surfactin (C_14_)
	1102.7	[M + K]^+^	Surfactin (C_15_)

### 3.5 *Bacillus velezensis* LT1 significantly changed the mycelial morphology of *Sclerotium rolfsii* LC1

**Figure 3:**
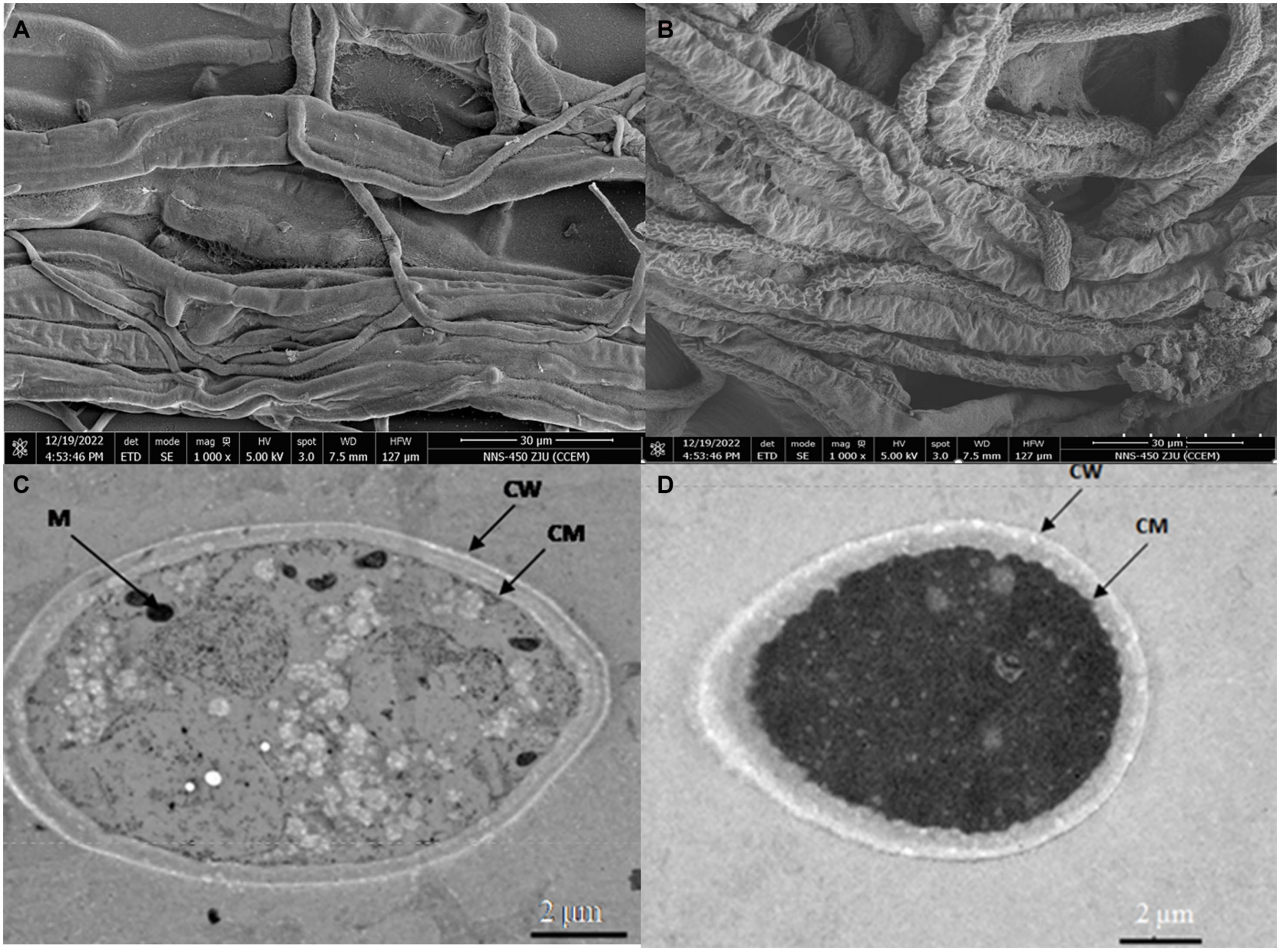
SEM and TEM images of mycelial morphology of *S. rolfsii* LC1. **(A)** SEM image of normal *S. rolfsii* LC1 mycelia; **(B)** SEM image of *S. rolfsii* LC1 mycelia under *B. velezensis* LT1 stress; **(C)** TEM image of normal *S. rolfsii* LC1 mycelia; **(D)** TEM image of *S. rolfsii* LC1 mycelia under *B. velezensis* LT1 stress. CM: cell membrane; CW: cell wall; M: mitochondria.

To further explore the effect of *B. velezensis* LT1 on *S. rolfsii* LC1, we observed the mycelial morphology of *S. rolfsii* LC1 under *B. velezensis* LT1 stress using scanning electron microscopy (SEM) and transmission electron microscopy (TEM). SEM observations showed that normal mycelia were arranged in an orderly manner with a smooth surface, uniform thickness, good extensibility, and a complete cell wall (Figure 3A). However, under the stress of *B. velezensis* LT1, the mycelia of *S. rolfsii* LC1 were folded with partial mycelial rupture, cytoplasmic leakage, and mycelial fragmentation (Figure 3B). Transmission electron microscopy showed that the control mycelia grew normally, with uniform and complete cell walls, clear boundaries, light cytoplasm, uniform distribution, clear nuclei, abundant and complete organelles evenly distributed in the cytoplasm, and a clear structure (Figure 3C). After treatment with *B. velezensis* LT1, the mycelial cell walls were significantly thickened, the cytoplasm was concentrated and dark in color, and a large number of white particles appeared. The nuclear structure was unclear, and no obvious nuclear membrane boundary was observed. Additionally, the number of mitochondria was significantly reduced (Figure 3D). These observations suggest that *B. velezensis* LT1 may have a destructive effect on the mycelial structure of *S. rolfsii* LC1.

## 4 Discussion

The use of biological control agents (BCAs) is an important means to reduce the amount of chemical fungicides and protect the ecological environment (Bonaterra et al., 2022). *Bacillus* species are the most common soil-beneficial bacteria and one of the most studied and widely used BCAs (Kovács, 2019). In this study, *B. velezensis* LT1 was isolated from the rhizosphere soil where southern blight occurred severely. It can produce a variety of lipopeptide antagonistic fungal substances to inhibit the mycelial growth of *S. rolfsii* LC, a pathogen that causes southern blight. In pot assays, *B. velezensis* LT1 also showed beneficial control efficacy of southern blight. At the same time, *B. velezensis* LT1 had a strong inhibitory effect on the mycelial growth of various pathogens, showing great potential for biocontrol. This is the first time that *B. velezensis* has been reported to be used in the prevention and control of diseases in Chinese herbal medicines.

*B. velezensis* was first isolated, identified, and officially named by Ruiz et al. from the Velez River in 2005 (Ruiz-García et al., 2005). In 2007, the whole genome of *B. velezensis* FZB42 was sequenced, revealing the presence of nine gene clusters that encode antimicrobial compounds. These nine gene clusters, namely, *fen*, *srf*, *bac*, *mln*, *bae*, *dfn*, *dhb*, *bmy*, and *nrs*, encode synthase for the biosynthesis of antimicrobial compounds, such as fengycins, surfactin, bacillomycin, and others (Chen et al., 2007). Multiple studies have reported that *B. velezensis* produces various antibacterial compounds against plant pathogens. Vahidinasab et al. reported that *B. velezensis* UTB96 produced surfactin, fengycin, and iturin, which showed excellent inhibitory activity against the soybean fungal pathogen *Diaporthe longicolla*. Lysine and alanine could stimulate the production of these three lipopeptide antibacterial compounds (Vahidinasab et al., 2022). Kim et al. isolated *B. velezensis* TSA32-1 from the soil, which could inhibit the growth of *F. graminearum*, *F. fujikuroi*, *A. alternatia*, and *Diaporthe actinidiae*. The genome analysis of the strain TSA32-1 revealed the presence of a synthetic gene cluster of bacillaene and surfactant (Kim et al., 2022). In addition, *B. velezensis* CE100 secretes macrolactin A, which effectively controls the occurrence of bacterial diseases, such as bacterial soft rot of cucumber caused by *Pectobacterium carotovorum* (Htwe Maung et al., 2022). In this study, the lipopeptide antibiotics isolated from the fermentation filtrate of *B. velezensis* LT1 were found to be similar to those reported in previous studies. However, *B. velezensis* LT1 produced a relatively large number of antimicrobial compounds, including bacillomycin, surfactin, iturin, and fengycin, with different carbon chain lengths. Notably, the types of iturin and fengycin produced by *B. velezensis* LT1 were much higher than those reported in previous studies. These results suggest that *B. velezensis* LT1 may inhibit the growth of *S. rolfsii* LC1 by producing lipopeptide antibacterial compounds. However, this study did not differentiate the inhibitory effects of different lipopeptide antibacterial compounds on *S. rolfsii* LC1 or identify the main antibacterial compounds responsible for inhibiting its growth. Furthermore, the study did not explore the specific mechanism of action of lipopeptide antibacterial compounds on *S. rolfsii* LC1. Future research should focus on identifying the main antibacterial compounds responsible for inhibiting *S. rolfsii* LC1 and clarifying their molecular mechanisms of action. In addition, *B. velezensis* has been found to promote plant growth by secreting siderophores, indole-3-acetic acid (IAA), and volatile organic compounds (Raza et al., 2016; Myo et al., 2019; Wang et al., 2020). For example, *B. velezensis* HNH9 promoted the growth of upland cotton plants by upregulating the expression of growth-linked genes, such as *EXP6*, *ARF1*, *ARF18*, *IAA9*, *CKX6*, and *GID1b* (Hasan et al., 2022). Similarly, *B. velezensis* SX13 has been shown to promote the uptake and transport of elements by improving root structure and upregulating *CsNRT1* expression, resulting in increased rates of element uptake and transport, which, in turn, promote photosynthesis and the activity of enzymes associated with carbon and nitrogen metabolism (Wang et al., 2022). Another study found that *B. velezensis* FH-1 promoted rice growth by regulating rhizosphere microbiome structure and nitrification function (Wang et al., 2023). In this study, the slow seed germination and growth of *C. chinensis* prevented determining whether *B. velezensis* LT1 could promote its growth. Nevertheless, the effect of *B. velezensis* LT1 on the germination of wheat seeds and rooting of onion was tested using hydroponics, and the results indicated that it could promote their growth (data not shown). This suggests that *B. velezensis* LT1 has the potential to promote plant growth. In future research, we will also investigate the promotion and mechanism of *B. velezensis* LT1 on the growth and accumulation of effective medicinal components of *C. chinensis*. In the past decade, researchers have discovered that complex microbial community assemblages in the rhizosphere, rather than single microbial strains, play an essential role in protecting plants from pathogens. Multiomics sequencing technologies have been widely used in the study of plant microbiome (Ritpitakphong et al., 2016). Therefore, the construction of synthetic communities (SynComs) based on beneficial microorganisms for plant disease control and growth promotion has gradually become a research focus. Schmitz et al. isolated the root core bacterial microflora of the desert plant *Indigofera argentea* and constructed a SynCom composed of 15 strains. This SynCom showed a strong growth-promoting effect on tomatoes under salt stress (Schmitz et al., 2022). Li et al. isolated bacterial strains from *Astragalus mongholicus* infected with *F. oxysporum* and constructed a SynCom that included *Stenotrophomonas* sp., *Rhizobium* sp., *Ochrobactrum* sp., and *Advenella* sp. The simplified SynCom effectively reduced the occurrence of root rot caused by *F. oxysporum* on *A. mongholicus* (Li et al., 2021). The use of cross-kingdom (fungal and bacterial) SynComs was more effective in suppressing Fusarium wilt disease (FWD) of tomato than fungal or bacterial SynComs alone (Zhou et al., 2022). The results demonstrate the great potential of SynComs in controlling crop diseases and promoting crop growth. In this study, more than 30 different strains of bacteria from 24 genera and 4 phyla were isolated from rhizosphere soil with severe rhizome rot of *C. chinensis*. Among them, eight strains could directly inhibit the growth of *S. rolfsii* LC1. Additionally, *Pseudomonas* sp., *Streptomyces* sp., *Microbacterium* sp., and *Pseudarthrobacter* sp. have often been reported as beneficial microorganisms in plants (Vurukonda et al., 2018; Zhao et al., 2019; Ham et al., 2022; Venturi, 2022). These beneficial bacteria can serve as an important foundation for constructing SynComs to control root rot in *C. chinensis*.

Many *Bacillus* species have been reported to act as biocontrol agents for plant diseases (Ribeiro et al., 2021; Nie et al., 2022; Baard et al., 2023; Hernández-Huerta et al., 2023; Kadiri et al., 2023). According to previous reports, lipopeptide antimicrobial compounds play a key role in *Bacillus* to inhibit of the growth and development of plant pathogens (Amaning Danquah et al., 2022; Kim et al., 2022). Similarly, *B. velezensis* LT1, isolated from the rhizosphere soil with severe rhizome rot of *C. chinensis*, can inhibit the growth of many plant pathogens by secreting lipopeptide antimicrobial compounds. The great biocontrol potential of *B. velezensis* LT1 deserves further detailed study. The potential of lipopeptide antimicrobial compounds identified in this study for plant disease control should also be further investigated. Bacterial isolates that stimulate plant growth and inhibit the development of fungal pathogens would be good candidates for the development of bioactive formulations.

In summary, our findings demonstrate the ability of *B. velezensis* LC1 to inhibit the growth of harmful fungi and present the novel use of *B. velezensis* LC1 as a means of controlling southern blight in *C. chinensis* (Graphical Abstract). These results have significant implications as southern blight has already inflicted substantial harm on *C. chinensis* cultivation in China and continues to spread.

## Data availability statement

The original contributions presented in the study are included in the article/Supplementary material, further inquiries can be directed to the corresponding authors.

## Author contributions

TT: Conceptualization, Data curation, Visualization, Writing – original draft. FW: Data curation, Investigation, Writing – original draft. HH: Data curation, Writing – original draft. JG: Data curation, Investigation, Writing – original draft. XG: Funding acquisition, Investigation, Writing – original draft. YD: Conceptualization, Data curation, Writing – original draft. XW: Data curation, Visualization, Writing – original draft. QW: Writing – review & editing. JY: Visualization, Writing – review & editing.

## Footnotes

^1^ https://www.ncbi.nlm.nih.gov/
