# Supplementary material for: Bacillus velezensis LT1: a potential biocontrol agent for southern blight on Coptis chinensis
Source: Front Microbiol. 2024 Mar 4;15:1337655. doi: 10.3389/fmicb.2024.1337655 (PMC10946422; doi:10.3389/fmicb.2024.1337655)
Supplement: Supplementary file 1 [file Data_Sheet_1.docx]

>Bacillus velezensis LT1

GCCTATTACATGCAAGTCGAGCGGACAGATGGGAGCTTGCTCCCTGATGTTAGCGGCGGACGGGTGAGTAACACGTGGGTAACCTGCCTGTAAGACTGGGATAACTCCGGGAAACCGGGGCTAATACCGGATGGTTGTTTGAACCGCATGGTTCAGACATAAAAGGTGGCTTCGGCTACCACTTACAGATGGACCCGCGGCGCATTAGCTAGTTGGTGAGGTAACGGCTCACCAAGGCGACGATGCGTAGCCGACCTGAGAGGGTGATCGGCCACACTGGGACTGAGACACGGCCCAGACTCCTACGGGAGGCAGCAGTAGGGAATCTTCCGCAATGGACGAAAGTCTGACGGAGCAACGCCGCGTGAGTGATGAAGGTTTTCGGATCGTAAAGCTCTGTTGTTAGGGAAGAACAAGTGCCGTTCAAATAGGGCGGCACCTTGACGGTACCTAACCAGAAAGCCACGGCTAACTACGTGCCAGCAGCCGCGGTAATACGTAGGTGGCAAGCGTTGTCCGGAATTATTGGGCGTAAAGGGCTCGCAGGCGGTTTCTTAAGTCTGATGTGAAAGCCCCCGGCTCAACCGGGGAGGGTCATTGGAAACTGGGGAACTTGAGTGCAGAAGAGGAGAGTGGAATTCCACGTGTAGCGGTGAAATGCGTAGAGATGTGGAGGAACACCAGTGGCGAAGGCGACTCTCTGGTCTGTAACTGACGCTGAGGAGCGAAAGCGTGGGGAGCGAACAGGATTAGATACCCTGGTAGTCCACGCCGTAAACGATGAGTGCTAAGTGTTAGGGGGTTTCCGCCCCTTAGTGCTGCAGCTAACGCATTAAGCACTCCGCCTTGGGGAGTACGGTCCGCAAGACTGAAACTCCAAGGGATTTGACGGGGGGCCCGCACAAGCGGGGGGAGCTTGGGGTTTAATTCAAAGCAACGGCGAAGAACCTTAACCAAGGCTTTGACTTTCTTCTGG

>Bacillus zanthoxyli LT2

TGCAGTCGAGCGACTGATTAGAAGCTTGCTTCTATGACGTTAGCGGCGGACGGGTGAGTAACACGTGGGCAACCTGCCTGTAAGACTGGGATAACTTCGGGAAACCGAAGCTAATACCGGATAGGATCTTCTCCTTCATGGGAGATGATTGAAAGATGGTTTCGGCTATCACTTACAGATGGGCCCGCGGTGCATTAGCTAGTTGGTGAGGTAACGGCTCACCAAGGCAACGATGCATAGCCGACCTGAGAGGGTGATCGGCCACACTGGGACTGAGACACGGCCCAGACTCCTACGGGAGGCAGCAGTAGGGAATCTTCCGCAATGGACGAAAGTCTGACGGAGCAACGCCGCGTGAGTGATGAAGGCTTTCGGGTCGTAAAACTCTGTTGTTAGGGAAGAACAAGTACGAGAGTAACTGCTCGTACCTTGACGGTACCTAACCAGAAAGCCACGGCTAACTACGTGCCAGCAGCCGCGGTAATACGTAGGTGGCAAGCGTTATCCGGAATTATTGGGCGTAAAGCGCGCGCAGGCGGTTTCTTAAGTCTGATGTGAAAGCCCACGGCTCAACCGTGGAGGGTCATTGGAAACTGGGGAACTTGAGTGCAGAAGAGAAAAGCGGAATTCCACGTGTAGCGGTGAAATGCGTAGAGATGTGGAGGAACACCAGTGGCGAAGGCGGCTTTTTGGTCTGTAACTGACGCTGAGGCGCGAAAGCGTGGGGAGCAAACAGGATTAGATACCCTGGTAGTCCACGCCGTAAACGATGAGTGCTAAGTGTTAGAGGGTTTCCGCCCTTTAGTGCTGCAGCTAACGCATTAAGCACTCCGCCTGGGGAGTACGGTCGCAAGACTGAAACTCAAAGGAATTGACGGGGGCCCGCACAAGCGGTGGAGCATGTGGTTTAATTCGAAGCAACGCGAAGAACCTTACCAGGTCTTGACATCCTCTGACAACTCTAGAGATAGAGCGTTCCCCTTCGGGGGACAGAGTGACAGGTGGTGCATGGTTGTCGTCAGCTCGTGTCGTGAGATGTTGGGTTAAGTCCCGCAACGAGCGCAACCCTTGATCTTAGTTGCCAGCATTTAGTTGGGCACTCTAAGGTGACTGCCGGTGACAAACCGGAGGAAGGTGGGGATGACGTCAAATCATCATGCCCCTTATGACCTGGGCTACACACGTGCTACAATGGATGGTACAAAGGGCTGCAAGACCGCGAGGTCAAGCCAATCCCATAAAACCATTCTCAGTTCGGATTGTAGGCTGCAACTCGCCTACATGAAGCTGGAATCGCTAGTAATCGCGGATCAGCATGCCGCGGTGAATACGTTCCCGGGCCTTGTACACACCGCCCGTCACACCACGAGAGTTTGAACACCCGAAGTCGGTGGAGTAACCGTAAGGAG

>Rossellomorea oryzaecorticis LT3

TGCAGTCGAGCGGATCGATGGGAGCTTGCTCCCTGAGATTAGCGGCGGACGGGTGAGTAACACGTGGGCAACCTGCCTATAAGACTGGGATAACTTCGGGAAACCGGAGCTAATACCGGATACGTTCTTTTCTCGCATGAGAGAAGATGGAAAGACGGTTTACGCTGTCACTTATAGATGGGCCCGCGGCGCATTAGCTAGTTGGTGAGGTAATGGCTCACCAAGGCGACGATGCGTAGCCGACCTGAGAGGGTGATCGGCCACACTGGGACTGAGACACGGCCCAGACTCCTACGGGAGGCAGCAGTAGGGAATCTTCCGCAATGGACGAAAGTCTGACGGAGCAACGCCGCGTGAACGAAGAAGGCCTTCGGGTCGTAAAGTTCTGTTGTTAGGGAAGAACAAGTACCAGAGTAACTGCTGGTACCTTGACGGTACCTAACCAGAAAGCCACGGCTAACTACGTGCCAGCAGCCGCGGTAATACGTAGGTGGCAAGCGTTGTCCGGAATTATTGGGCGTAAAGCGCGCGCAGGTGGTTCTTTAAGTCTGATGTGAAAGCCCACGGCTCAACCGTGGAGGGTCATTGGAAACTGGGGAACTTGAGTGCAGAAGAGGAAAGTGGAATTCCAAGTGTAGCGGTGAAATGCGTAGAGATTTGGAGGAACACCAGTGGCGAAGGCGACTTTCTGGTCTGTAACTGACACTGAGGCGCGAAAGCGTGGGGAGCAAACAGGATTAGATACCCTGGTAGTCCACGCCGTAAACGATGAGTGCTAAGTGTTAGAGGGTTTCCGCCCTTTAGTGCTGCAGCTAACGCATTAAGCACTCCGCCTGGGGAGTACGGCCGCAAGGCTGAAACTCAAAGGAATTGACGGGGGCCCGCACAAGCGGTGGAGCATGTGGTTTAATTCGAAGCAACGCGAAGAACCTTACCAGGTCTTGACATCCTCTGACAACCCTAGAGATAGGGCTTTCCCCTTCGGGGGACAGAGTGACAGGTGGTGCATGGTTGTCGTCAGCTCGTGTCGTGAGATGTTGGGTTAAGTCCCGCAACGAGCGCAACCCTTGATCTTAGTTGCCAGCATTCAGTTGGGCACTCTAAGGTGACTGCCGGTGACAAACCGGAGGAAGGTGGGGATGACGTCAAATCATCATGCCCCTTATGACCTGGGCTACACACGTGCTACAATGGATGGTACAAAGGGCTGCAAACCTGCGAAGGTAAGCGAATCCCATAAAGCCATTCTCAGTTCGGATTGCAGGCTGCAACTCGCCTGCATGAAGCCGGAATCGCTAGTAATCGCGGATCAGCATGCCGCGGTGAATACGTTCCCGGGCCTTGTACACACCGCCCGTCACACCACGAGAGTTTGAACACCCGAAGTCGGTGAGGTAACCTTATGGAGCCAGCCGCCTAA

>Chryseobacterium indologenes LT4

GTTGCAGCCGAGCGGTAGAGATTCTTCGGAATCTTGAGAGCGGCGTACGGGTGCGGAACACGTGTGCAACCTGCCTTTATCTGGGGGATAGCCTTTCGAAAGGAAGATTAATACCCCATAATATATTGAATGGCATCATTTGATATTGAAAACTCCGGTGGATAGAGATGGGCACGCGCAAGATTAGATAGTTGGTGAGGTAACGGCTCACCAAGTCTACGATCTTTAGGGGGCCTGAGAGGGTGATCCCCCACACTGGTACTGAGACACGGACCAGACTCCTACGGGAGGCAGCAGTGAGGAATATTGGACAATGGGTGAGAGCCTGATCCAGCCATCCCGCGTGAAGGACGACGGCCCTATGGGTTGTAAACTTCTTTTGTATAGGGATAAACCTAGATACGTGTATCTAGCTGAAGGTACTATACGAATAAGCACCGGCTAACTCCGTGCCAGCAGCCGCGGTAATACGGAGGGTGCAAGCGTTATCCGGATTTATTGGGTTTAAAGGGTCCGTAGGCGGATTTGTAAGTCAGTGGTGAAATCTCACAGCTTAACTGTGAAACTGCCATTGATACTGCAAGTCTTGAGTGTTGTTGAAGTAGCTGGAATAAGTAGTGTAGCGGTGAAATGCATAGATATTACTTAGAACACCAATTGCGAAGGCAGGTTACTAAGCAACAACTGACGCTGATGGACGAAAGCGTGGGGAGCGAACAGGATTAGATACCCTGGTAGTCCACGCCGTAAACGATGCTAACTCGTTTTTGGGCTTTCGGGTTCAGAGACTAAGCGAAAGTGATAAGTTAGCCACCTGGGGAGTACGAACGCAAGTTTGAAACTCAAAGGAATTGACGGGGGCCCGCACAAGCGGTGGATTATGTGGTTTAATTCGATGATACGCGAGGAACCTTACCAAGGCTTAAATGGGAAATGACAGGTTTAGAAATAGACTTTTCTTCGGACATTTTTCAAGGTGCTGCATGGTTGTCGTCAGCTCGTGCCGTGAGGTGTTAGGTTAAGTCCTGCAACGAGCGCAACCCCTGTCACTAGTTGCCATCATTAAGTTGGGGACTCTAGTGAGACTGCCTACGCAAGTAGAGAGGAAGGTGGGGATGACGTCAAATCATCACGGCCCTTACGCCTTGGGCCACACACGTAATACAATGGCCGGTACAGAGGGCAGCTACACTGCGAAGTGATGCAAATCTCGAAAGCCGGTCTCAGTTCGGATTGGAGTCTGCAACTCGACTCTATGAAGCTGGAATCGCTAGTAATCGCGCATCAGCCATGGCGCGGTGAATACGTTCCCGGGCCTTGTACACACCGCCCGTCAAGCCATGGAAGTCTGGGGTACCTGAAGTCGGTGACCGTAACAGAGCTT

>Pseudomonas rhizosphaerae LT5

GTCCCCCCGAAGGTTAGACTAGCTACTTCTGGTGCAACCCACTCCCATGGTGTGACGGGCGGTGTGTACAAGGCCCGGGAACGTATTCACCGCGACATTCTGATTCGCGATTACTAGCGATTCCGACTTCACGCAGTCGAGTTGCAGACTGCGATCCGGACTACGATCGGTTTTATGGGATTAGCTCCACCTCGCGGCTTGGCAACCCTTTGTACCGACCATTGTAGCACGTGTGTAGCCCAGGCCGTAAGGGCCATGATGACTTGACGTCATCCCCACCTTCCTCCGGTTTGTCACCGGCAGTCTCCTTAGAGTGCCCACCATAACGTGCTGGTAACTAAGGACAAGGGTTGCGCTCGTTACGGGACTTAACCCAACATCTCACGACACGAGCTGACGACAGCCATGCAGCACCTGTCTCAATGTTCCCGAAGGCACCCATCCATCTCTGGAAAGTTCATTGGATGTCAAGGCCTGGTAAGGTTCTTCGCGTTGCTTCGAATTAAACCACATGCTCCACCGCTTGTGCGGGCCCCCGTCAATTCATTTGAGTTTTAACCTTGCGGCCGTACTCCCCAGGCGGTCAACTTAATGCGTTAGCTGCGCCACTAAAATCTCAAGGATTCCAACGGCTAGTTGACATCGTTTACGGCGTGGACTACCAGGGTATCTAATCCTGTTTGCTCCCCACGCTTTCGCACCTCAGTGTCAGTATGAGCCCAGGTGGTCGCCTTCGCCACTGGTGTTCCTTCCTATATCTACGCATTTCACCGCTACACAGGAAATTCCACCACCCTCTGCCCTACTCTAGCTTGCCAGTTTTGGATGCAGTTCCCAGGTTGAGCCCGGGGCTTTCACATCCAACTTAACAAACCACCTACGCGCGCTTTACGCCCAGTAATTCCGATTAACGCTTGCACCCTCTGTATTACCGCGGCTGCTGGCACAGAGTTAGCCGGTGCTTATTCTGTCGGTAACGTCAAAACAGCAAGGTATTCGCTTACTGCCCTTCCTCCCAACTTAAAGTGCTTTACAATCCGAAGACCTTCTTCACACACGCGGCATGGCTGGATCAGGCTTTCGCCCATTGTCCAATATTCCCCACTGCTGCCTCCCGTAGGAGTCTGGACCGTGTCTCAGTTCCAGTGTGACTGATCATCCTCTCAGACCAGTTACGGATCGTCGCCTTGGTGAGCCATTACCTCACCAACTAGCTAATCCGACCTAGGCTCATCTGATAGCGCAAGGCCCGAAGGTCCCCTGCTTTCTCCCGTAGGACGTATGCGGTATTAGCGTTCCTTTCGAAACGTTGTCCCCCACTACCAGGCAGATTCCTAGGCATTACTCACCCGTCCGCCGCTGAATCAGAGAGCAAGCTCCCTTCATCCGCTCGACTGCA

>Chryseobacterium sp LT6

AAGCTCTGTTACGGTCACCGACTTCAGGTACCCCAGACTTCCATGGCTTGACGGGCGGTGTGTACAAGGCCCGGGAACGTATTCACCGCGCCATGGCTGATGCGCGATTACTAGCGATTCCAGCTTCATAGAGTCGAGTTGCAGACTCCAATCCGAACTGAGACCGGCTTTCGAGATTTGCATCACTTCGCAGTGTAGCTGCCCTCTGTACCGGCCATTGTATTACGTGTGTGGCCCAAGGCGTAAGGGCCGTGATGATTTGACGTCATCCCCACCTTCCTCTCTACTTGCGTAGGCAGTCTCACTAGAGTCCCCAACTTAATGATGGCAACTAGTGACAGGGGTTGCGCTCGTTGCAGGACTTAACCTAACACCTCACGGCACGAGCTGACGACAACCATGCAGCACCTTGAAAAATGTCCGAAGAAAAGTCTATTTCTAAACCTGTCATTTCCCATTTAAGCCTTGGTAAGGTTCCTCGCGTATCATCGAATTAAACCACATAATCCACCGCTTGTGCGGGCCCCCGTCAATTCCTTTGAGTTTCAAACTTGCGTTCGTACTCCCCAGGTGGCTAACTTATCACTTTCGCTTAGTCTCTGAACCCGAAAGCCCAAAAACGAGTTAGCATCGTTTACGGCGTGGACTACCAGGGTATCTAATCCTGTTCGCTCCCCACGCTTTCGTCCATCAGCGTCAGTTGTTGCTTAGTAACCTGCCTTCGCAATTGGTGTTCTAAGTAATATCTATGCATTTCACCGCTACACTACTTATTCCAGCTACTTCAACAACACTCAAGACTTGCAGTATCAATGGCAGTTTCACAGTTAAGCTGTGAGATTTCACCACTGACTTACAAATCCGCCTACGGACCCTTTAAACCCAATAAATCCGGATAACGCTTGCACCCTCCGTATTACCGCGGCTGCTGGCACGGAGTTAGCCGGTGCTTATTCGTATAGTACCTTCAGCTAGATACACGTATCTAGGTTTATCCCTATACAAAAGAAGTTTACAACCCATAGGGCCGTCGTCCTTCACGCGGGATGGCTGGATCAGGCTCTCACCCATTGTCCAATATTCCTCACTGCTGCCTCCCGTAGGAGTCTGGTCCGTGTCTCAGTACCAGTGTGGGGGATCACCCTCTCAGGCCCCCTAAAGATCGTAGACTTGGTGAGCCGTTACCTCACCAACTATCTAATCTTGCGCGTGCCCATCTCTATCCACCGGAGTTTTCAATATCAAATGATGCCATTCAATATATTATGGGGTATTAATCTTCCTTTCGAAAGGCTATCCCCCAGATAAAGGCAGGTTGCACACGTGTTCCGCACCCGTACGCCGCTCTCAAGACTCCGAAGACATCTCTACCGCTCCGGCTGC

>Chryseobacterium vietnamense LT7

CGGTAGAGATTCTTCGGAGTCTTGAGAGCGGCGTACGGGTGCGGAACACGTGTGCAACCTGCCTTTATCTGGGGGATAGCCTTTCGAAAGGAAGATTAATACCCCATAATATATTGAATGGCATCATTTGATATTGAAAACTCCGGTGGATAGAGATGGGCACGCGCAAGATTAGATAGTTGGTGAGGTAACGGCTCACCAAGTCTACGATCTTTAGGGGGCCTGAGAGGGTGATCCCCCACACTGGTACTGAGACACGGACCAGACTCCTACGGGAGGCAGCAGTGAGGAATATTGGACAATGGGTGAGAGCCTGATCCAGCCATCCCGCGTGAAGGACGACGGCCCTATGGGTTGTAAACTTCTTTTGTATAGGGATAAACCTAGATACGTGTATCTAGCTGAAGGTACTATACGAATAAGCACCGGCTAACTCCGTGCCAGCAGCCGCGGTAATACGGAGGGTGCAAGCGTTATCCGGATTTATTGGGTTTAAAGGGTCCGTAGGCGGATTTGTAAGTCAGTGGTGAAATCTCACAGCTTAACTGTGAAACTGCCATTGATACTGCAAGTCTTGAGTGTTGTTGAAGTAGCTGGAATAAGTAGTGTAGCGGTGAAATGCATAGATATTACTTAGAACACCAATTGCGAAGGCAGGTTACTAAGCAACAACTGACGCTGATGGACGAAAGCGTGGGGAGCGAACAGGATTAGATACCCTGGTAGTCCACGCCGTAAACGATGCTAACTCGTTTTTGGGCTTTCGGGTTCAGAGACTAAGCGAAAGTGATAAGTTAGCCACCTGGGGAGTACGAACGCAAGTTTGAAACTCAAAGGAATTGACGGGGGCCCGCACAAGCGGTGGATTATGTGGTTTAATTCGATGATACGCGAGGAACCTTACCAAGGCTTAAATGGGAAATGACAGGTTTAGAAATAGACTTTTCTTCGGACATTTTTCAAGGTGCTGCATGGTTGTCGTCAGCTCGTGCCGTGAGGTGTTAGGTTAAGTCCTGCAACGAGCGCAACCCCTGTCACTAGTTGCCATCATTAAGTTGGGGACTCTAGTGAGACTGCCTACGCAAGTAGAGAGGAAGGTGGGGATGACGTCAAATCATCACGGCCCTTACGCCTTGGGCCACACACGTAATACAATGGCCGGTACAGAGGGCAGCTACACTGCGAAGTGATGCAAATCTCGAAAGCCGGTCTCAGTTCGGATTGGAGTCTGCAACTCGACTCTATGAAGCTGGAATCGCTAGTAATCGCGCATCAGCCATGGCGCGGTGAATACGTTCCCGGGCCTTGTACACACCGCCCGTCAAGCCATGGAAGTCTGGGGTACCTGAAGTCGGTGACCGGTAACAGGAGCTGCCTA

>Streptomyces zaomyceticus LT8

TCCCTCCCACAAGGGGTTGGGCCACCGGCTTCGGGTGTTACCGACTTTCGTGACGTGACGGGCGGTGTGTACAAGGCCCGGGAACGTATTCACCGCAGCAATGCTGATCTGCGATTACTAGCAACTCCGACTTCATGGGGTCGAGTTGCAGACCCCAATCCGAACTGAGACCGGCTTTTTGAGATTCGCTCCGCCTCGCGGCATCGCAGCTCTTTGTACCGGCCATTGTAGCACGTGTGCAGCCCAAGACATAAGGGGCATGATGACTTGACGTCGTCCCCACCTTCCTCCGAGTTGACCCCGGCGGTCTCCTGTGAGTCCCCATCACCCCGAAGGGCATGCTGGCAACACAGGACAAGGGTTGCGCTCGTTGCGGGACTTAACCCAACATCTCACGACACGAGCTGACGACAGCCATGCACCACCTGTATACCGACCACAAGGGGGGCACTATCTCTAATGCTTTCCGGTATATGTCAAGCCTTGGTAAGGTTCTTCGCGTTGCGTCGAATTAAGCCACATGCTCCGCTGCTTGTGCGGGCCCCCGTCAATTCCTTTGAGTTTTAGCCTTGCGGCCGTACTCCCCAGGCGGGGAACTTAATGCGTTAGCTGCGGCACCGACGACGTGGAATGTCGCCAACACCTAGTTCCCAACGTTTACGGCGTGGACTACCAGGGTATCTAATCCTGTTCGCTCCCCACGCTTTCGCTCCTCAGCGTCAGTAATGGCCCAGAGATCCGCCTTCGCCACCGGTGTTCCTCCTGATATCTGCGCATTTCACCGCTACACCAGGAATTCCGATCTCCCCTACCACACTCTAGCCTGCCCGTATCGGATGCAGACCCGGGGTTAAGCCCCGGGCTTTCACACCCGACGTGACAAGCCGCCTACGAGCTCTTTACGCCCAATAATTCCGGACAACGCTTGCGCCCTACGTATTACCGCGGCTGCTGGCACGTAGTTAGCCGGCGCTTCTTCTGCAGGTACCGTCACTTTCGCTTCTTCCCTGCTGAAAGAGGTTTACAACCCGAAGGCCGTCATCCCTCACGCGGCGTCGCTGCATCAGGCTTTCGCCCATTGTGCAATATTCCCCACTGCTGCCTCCCGTAGGAGTCTGGGCCGTGTCTCAGTCCCAGTGTGGCCGGTCGCCCTCTCAGGCCGGCTACCCGTCGTCGCCTTGGTAGGCCATTACCCCACCAACAAGCTGATAGGCCGCGGGCTCATCCTTCACCGCCGGAGCTTTTAACCAGCTCCCATGCGGAAGCCGGTGTTATCCGGTATTAGACCCCGTTTCCAGGGCTTGTCCCAGAGTGAAGGGCAGATTGCCCACGTGTTACTCACCCGTTCGCCACTAATCCACCCCGAAGGGCTTCATCGTTCGACTGCA

>Bacillus megaterium SLT1

CTATACTGCAGTCGAGCGACTGATTAGAAGCTTGCTTCTATGACGTTAGCGGCGGACGGGTGAGTAACACGTGGGCAACCTGCCTGTAAGACTGGGATAACTTCGGGAAACCGAAGCTAATACCGGATAGGATCTTCTCCTTCATGGGAGATGATTGAAAGATGGTTTCGGCTATCACTTACAGATGGGCCCGCGGTGCATTAGCTAGTTGGTGAGGTAACGGCTCACCAAGGCAACGATGCATAGCCGACCTGAGAGGGTGATCGGCCACACTGGGACTGAGACACGGCCCAGACTCCTACGGGAGGCAGCAGTAGGGAATCTTCCGCAATGGACGAAAGTCTGACGGAGCAACGCCGCGTGAGTGATGAAGGCTTTCGGGTCGTAAAACTCTGTTGTTAGGGAAGAACAAGTACAAGAGTAACTGCTTGTACCTTGACGGTACCTAACCAGAAAGCCACGGCTAACTACGTGCCAGCAGCCGCGGTAATACGTAGGTGGCAAGCGTTATCCGGAATTATTGGGCGTAAAGCGCGCGCAGGCGGTTTCTTAAGTCTGATGTGAAAGCCCACGGCTCAACCGTGGAGGGTCATTGGAAACTGGGGAACTTGAGTGCAGAAGAGAAAAGCGGAATTCCACGTGTAGCGGTGAAATGCGTAGAGATGTGGAGGAACACCAGTGGCGAAGGCGGCTTTTTGGTCTGTAACTGACGCTGAGGCGCGAAAGCGTGGGGAGCAAACAGGATTAGATACCCTGGTAGTCCACGCCGTAAACGATGAGTGCTAAGTGTTAGAGGGTTTCCGCCCTTTAGTGCTGCAGCTAACGCATTAAGCACTCCGCCTGGGGAGTACGGTCGCAAGACTGAAACTCAAAGGAATTGACGGGGGCCCGCACAAGCGGTGGAGCATGTGGTTTAATTCGAAGCAACGCGAAGAACCTTACCAGGTCTTGACATCCTCTGACAACTCTAGAGATAGAGCGTTCCCCTTCGGGGGACAGAGTGACAGGTGGTGCATGGTTGTCGTCAGCTCGTGTCGTGAGATGTTGGGTTAAGTCCCGCAACGAGCGCAACCCTTGATCTTAGTTGCCAGCATTTAGTTGGGCACTCTAAGGTGACTGCCGGTGACAAACCGGAGGAAGGTGGGGATGACGTCAAATCATCATGCCCCTTATGACCTGGGCTACACACGTGCTACAATGGATGGTACAAAGGGCTGCAAGACCGCGAGGTCAAGCCAATCCCATAAAACCATTCTCAGTTCGGATTGTAGGCTGCAACTCGCCTACATGAAGCTGGAATCGCTAGTAATCGCGGATCAGCATGCCGCGGTGAATACGTTCCCGGGCCTTGTACACACCGCCCGTCACACCACGAGAGTTTGAACACCCGAAGTCGGGGAGTAACCGTAAGGAGCTAACCGCCTAAG

>Pseudomonas vancouverensis SLT2

GGTACCGTCCTCCCGAAGGTTAGACTAGCTACTTCTGGTGCAACCCACTCCCATGGTGTGACGGGCGGTGTGTACAAGGCCCGGGAACGTATTCACCGCGACATTCTGATTCGCGATTACTAGCGATTCCGACTTCACGCAGTCGAGTTGCAGACTGCGATCCGGACTACGATCGGTTTTGTGGGATTAGCTCCACCTCGCGGCTTGGCAACCCTCTGTACCGACCATTGTAGCACGTGTGTAGCCCAGGCCGTAAGGGCCATGATGACTTGACGTCATCCCCACCTTCCTCCGGTTTGTCACCGGCAGTCTCCTTAGAGTGCCCACCATAACGTGCTGGTAACTAAGGACAAGGGTTGCGCTCGTTACGGGACTTAACCCAACATCTCACGACACGAGCTGACGACAGCCATGCAGCACCTGTCTCAATGTTCCCGAAGGCACCAATCCATCTCTGGAAAGTTCATTGGATGTCAAGGCCTGGTAAGGTTCTTCGCGTTGCTTCGAATTAAACCACATGCTCCACCGCTTGTGCGGGCCCCCGTCAATTCATTTGAGTTTTAACCTTGCGGCCGTACTCCCCAGGCGGTCAACTTAATGCGTTAGCTGCGCCACTAAGAGCTCAAGGCTCCCAACGGCTAGTTGACATCGTTTACGGCGTGGACTACCAGGGTATCTAATCCTGTTTGCTCCCCACGCTTTCGCACCTCAGTGTCAGTATCAGTCCAGGTGGTCGCCTTCGCCACTGGTGTTCCTTCCTATATCTACGCATTTCACCGCTACACAGGAAATTCCACCACCCTCTACCATACTCTAGCTTGTCAGTTTTGAATGCAGTTCCCAGGTTGAGCCCGGGGCTTTCACATCCAACTTAACAAACCACCTACGCGCGCTTTACGCCCAGTAATTCCGATTAACGCTTGCACCCTCTGTATTACCGCGGCTGCTGGCACAGAGTTAGCCGGTGCTTATTCTGTCGGTAACGTCAAAATTGCAGAGTATTAATCTACAACCCTTCCTCCCAACTTAAAGTGCTTTACAATCCGAAGACCTTCTTCACACACGCGGCATGGCTGGATCAGGCTTTCGCCCATTGTCCAATATTCCCCACTGCTGCCTCCCGTAGGAGTCTGGACCGTGTCTCAGTTCCAGTGTGACTGATCATCCTCTCAGACCAGTTACGGATCGTCGCCTTGGTGAGCCATTACCTCACCAACTAGCTAATCCGACCTAGGCTCATCTGATAGCGCAAGGCCCGAAGGTCCCCTGCTTTCTCCCGTAGGACGTATGCGGTATTAGCGTCCCTTTCGAGACGTTGTCCCCCACTACCAGGCAGATTCCTAGGCATTACTCACCCGTCCGCCGCTGAATCAGGGAGCAAGCTCCCCTCATCCGCTCGACTGCAGGTAGG

> Pseudomonas vancouverensis SLT3

CTACCTGCAGTCGAGCGGATGAGGGGAGCTTGCTCCCTGATTCAGCGGCGGACGGGTGAGTAATGCCTAGGAATCTGCCTGGTAGTGGGGGACAACGTCTCGAAAGGGACGCTAATACCGCATACGTCCTACGGGAGAAAGCAGGGGACCTTCGGGCCTTGCGCTATCAGATGAGCCTAGGTCGGATTAGCTAGTTGGTGAGGTAATGGCTCACCAAGGCGACGATCCGTAACTGGTCTGAGAGGATGATCAGTCACACTGGAACTGAGACACGGTCCAGACTCCTACGGGAGGCAGCAGTGGGGAATATTGGACAATGGGCGAAAGCCTGATCCAGCCATGCCGCGTGTGTGAAGAAGGTCTTCGGATTGTAAAGCACTTTAAGTTGGGAGGAAGGGTTGTAGATTAATACTCTGCAATTTTGACGTTACCGACAGAATAAGCACCGGCTAACTCTGTGCCAGCAGCCGCGGTAATACAGAGGGTGCAAGCGTTAATCGGAATTACTGGGCGTAAAGCGCGCGTAGGTGGTTTGTTAAGTTGGATGTGAAAGCCCCGGGCTCAACCTGGGAACTGCATTCAAAACTGACAAGCTAGAGTATGGTAGAGGGTGGTGGAATTTCCTGTGTAGCGGTGAAATGCGTAGATATAGGAAGGAACACCAGTGGCGAAGGCGACCACCTGGACTGATACTGACACTGAGGTGCGAAAGCGTGGGGAGCAAACAGGATTAGATACCCTGGTAGTCCACGCCGTAAACGATGTCAACTAGCCGTTGGGAGCCTTGAGCTCTTAGTGGCGCAGCTAACGCATTAAGTTGACCGCCTGGGGAGTACGGCCGCAAGGTTAAAACTCAAATGAATTGACGGGGGCCCGCACAAGCGGTGGAGCATGTGGTTTAATTCGAAGCAACGCGAAGAACCTTACCAGGCCTTGACATCCAATGAACTTTCCAGAGATGGATTGGTGCCTTCGGGAACATTGAGACAGGTGCTGCATGGCTGTCGTCAGCTCGTGTCGTGAGATGTTGGGTTAAGTCCCGTAACGAGCGCAACCCTTGTCCTTAGTTACCAGCACGTTATGGTGGGCACTCTAAGGAGACTGCCGGTGACAAACCGGAGGAAGGTGGGGATGACGTCAAGTCATCATGGCCCTTACGGCCTGGGCTACACACGTGCTACAATGGTCGGTACAGAGGGTTGCCAAGCCGCGAGGTGGAGCTAATCCCACAAAACCGATCGTAGTCCGGATCGCAGTCTGCAACTCGACTGCGTGAAGTCGGAATCGCTAGTAATCGCGAATCAGAATGTCGCGGTGAATACGTTCCCGGGCCTTGTACACACCGCCCGTCACACCATGGGAGTGGGTGCACCAGAAGTAGCTAGTCTAAACCTTCGGGAGGACCGGTACC

> Pseudomonas putida SLT4

CTGATTCACCGTGGTACCGTCCCCCCGAAGGTTAGACTAGCTACTTCTGGTGCAACCCACTCCCATGGTGTGACGGGCGGTGTGTACAAGGCCCGGGAACGTATTCACCGCGACATTCTGATTCGCGATTACTAGCGATTCCGACTTCACGCAGTCGAGTTGCAGACTGCGATCCGGACTACGATCGGTTTTGTGGGATTAGCTCCACCTCGCGGCTTGGCAACCCTCTGTACCGACCATTGTAGCACGTGTGTAGCCCAGGCCGTAAGGGCCATGATGACTTGACGTCATCCCCACCTTCCTCCGGTTTGTCACCGGCAGTCTCCTTAGAGTGCCCACCATTACGTGCTGGTAACTAAGGACAAGGGTTGCGCTCGTTACGGGACTTAACCCAACATCTCACGACACGAGCTGACGACAGCCATGCAGCACCTGTCTCAATGTTCCCGAAGGCACCAATCCATCTCTGGAAAGTTCATTGGATGTCAAGGCCTGGTAAGGTTCTTCGCGTTGCTTCGAATTAAACCACATGCTCCACCGCTTGTGCGGGCCCCCGTCAATTCATTTGAGTTTTAACCTTGCGGCCGTACTCCCCAGGCGGTCAACTTAATGCGTTAGCTGCGCCACTAAAGACTCAAGGTCCCCAACGGCTAGTTGACATCGTTTACGGCGTGGACTACCAGGGTATCTAATCCTGTTTGCTCCCCACGCTTTCGCACCTCAGTGTCAGTATCAGTCCAGGTGGTCGCCTTCGCCACTGGTGTTCCTTCCTATATCTACGCATTTCACCGCTACACAGGAAATTCCACCACCCTCTACCATACTCTAGCTTGTCAGTTTTGAATGCAGTTCCCAGGTTGAGCCCGGGGCTTTCACATCCAACTTAACAAACCACCTACGCGCGCTTTACGCCCAGTAATTCCGATTAACGCTTGCACCCTCTGTATTACCGCGGCTGCTGGCACAGAGTTAGCCGGTGCTTATTCTGTCGGTAACGTCAAAACACTAACGTATTAGGTTAATGCCCTTCCTCCCAACTTAAAGTGCTTTACAATCCGAAGACCTTCTTCACACACGCGGCATGGCTGGATCAGGCTTTCGCCCATTGTCCAATATTCCCCACTGCTGCCTCCCGTAGGAGTCTGGACCGTGTCTCAGTTCCAGTGTGACTGATCATCCTCTCAGACCAGTTACGGATCGTCGCCTTGGTGAGCCATTACCTCACCAACTAGCTAATCCGACCTAGGCTCATCTGATAGCGCAAGGCCCGAAGGTCCCCTGCTTTCTCCCGTAGGACGTATGCGGTATTAGCGTTCCTTTCGAAACGTTGTCCCCCACTACCAGGCAGATTCCTAGGCATTACTCACCCGTCCGCCGCTGAATCAGAGAGCAAGCTCTCTTCATCCGCTCGACTGCAGGTAG

> Pseudomonas umsongensis SLT5

CCTACCTGCAAGTCGAGCGGATGACGAGAGCTTGCTCTCTGATTCAGCGGCGGACGGGTGAGTAATGCCTAGGAATCTGCCTGGTAGTGGGGGACAACGTTTCGAAAGGAACGCTAATACCGCATACGTCCTACGGGAGAAAGCAGGGGACCTTCGGGCCTTGCGCTATCAGATGAGCCTAGGTCGGATTAGCTAGTTGGTGAGGTAATGGCTCACCAAGGCGACGATCCGTAACTGGTCTGAGAGGATGATCAGTCACACTGGAACTGAGACACGGTCCAGACTCCTACGGGAGGCAGCAGTGGGGAATATTGGACAATGGGCGAAAGCCTGATCCAGCCATGCCGCGTGTGTGAAGAAGGTCTTCGGATTGTAAAGCACTTTAAGTTGGGAGGAAGGGCAGTAAATTAATACTTTGCTGTTTTGACGTTACCGACAGAATAAGCACCGGCTAACTCTGTGCCAGCAGCCGCGGTAATACAGAGGGTGCAAGCGTTAATCGGAATTACTGGGCGTAAAGCGCGCGTAGGTGGTTTGTTAAGTTGGATGTGAAAGCCCCGGGCTCAACCTGGGAACTGCATTCAAAACTGACAAGCTAGAGTATGGTAGAGGGTGGTGGAATTTCCTGTGTAGCGGTGAAATGCGTAGATATAGGAAGGAACACCAGTGGCGAAGGCGACCACCTGGACTGATACTGACACTGAGGTGCGAAAGCGTGGGGAGCAAACAGGATTAGATACCCTGGTAGTCCACGCCGTAAACGATGTCAACTAGCCGTTGGGAGCCTTGAGCTCTTAGTGGCGCAGCTAACGCATTAAGTTGACCGCCTGGGGAGTACGGCCGCAAGGTTAAAACTCAAATGAATTGACGGGGGCCCGCACAAGCGGTGGAGCATGTGGTTTAATTCGAAGCAACGCGAAGAACCTTACCAGGCCTTGACATCCAATGAACTTTCCAGAGATGGATTGGTGCCTTCGGGAACATTGAGACAGGTGCTGCATGGCTGTCGTCAGCTCGTGTCGTGAGATGTTGGGTTAAGTCCCGTAACGAGCGCAACCCTTGTCCTTAGTTACCAGCACGTTATGGTGGGCACTCTAAGGAGACTGCCGGTGACAAACCGGAGGAAGGTGGGGATGACGTCAAGTCATCATGGCCCTTACGGCCTGGGCTACACACGTGCTACAATGGTCGGTACAGAGGGTTGCCAAGCCGCGAGGTGGAGCTAATCCCACAAAACCGATCGTAGTCCGGATCGCAGTCTGCAACTCGACTGCGTGAAGTCGGAATCGCTAGTAATCGCGAATCAGAATGTCGCGGTGAATACGTTCCCGGGCCTTGTACACACCGCCCGTCACACCATGGGAGTGGGTTGCACCAGAAGTAGCTAGTCTAACCTTCGGGGGGACGGTACCAC

> Enterobacter sp SLT6

GGTAGCGCCCTCCCGAAGGGTTAAGCTACCTACTTCTTTTGCACCCACTCCCATGGTGTGACGGGCGGTGTGTACAAGGCCCGGGAACGTATTCACCGTAGCATTCTGATCTACGATTACTAGCGATTCCGACTTCATGGAGTCGAGTTGCAGACTCCAATCCGGACTACGACGCACTTTATGAGGTCCGCTTGCTCTCGCGAGGTCGCTTCTCTTTGTATGCGCCATTGTAGCACGTGTGTAGCCCTACTCGTAAGGGCCATGATGACTTGACGTCATCCCCACCTTCCTCCAGTTTATCACTGGCAGTCTCCTTTGAGTTCCCGGCCTAACCGCTGGCAACAAAGGATAAGGGTTGCGCTCGTTGCGGGACTTAACCCAACATTTCACAACACGAGCTGACGACAGCCATGCAGCACCTGTCTCAGAGTTCCCGAAGGCACCAAAGCATCTCTGCTAAGTTCTCTGGATGTCAAGAGTAGGTAAGGTTCTTCGCGTTGCATCGAATTAAACCACATGCTCCACCGCTTGTGCGGGCCCCCGTCAATTCATTTGAGTTTTAACCTTGCGGCCGTACTCCCCAGGCGGTCGACTTAACGCGTTAGCTCCGGAAGCCACGCCTCAAGGGCACAACCTCCAAGTCGACATCGTTTACGGCGTGGACTACCAGGGTATCTAATCCTGTTTGCTCCCCACGCTTTCGCACCTGAGCGTCAGTCTTTGTCCAGGGGGCCGCCTTCGCCACCGGTATTCCTCCAGATCTCTACGCATTTCACCGCTACACCTGGAATTCTACCCCCCTCTACAAGACTCTAGCCTGCCAGTTTCGAATGCAGTTCCCAGGTTGAGCCCGGGGATTTCACATCCGACTTGACAGACCGCCTGCGTGCGCTTTACGCCCAGTAATTCCGATTAACGCTTGCACCCTCCGTATTACCGCGGCTGCTGGCACGGAGTTAGCCGGTGCTTCTTCTGCGAGTAACGTCAATTGCTGTGCTTATTAACCACAACACCTTCCTCCTCGCTGAAAGTACTTTACAACCCGAAGGCCTTCTTCATACACGCGGCATGGCTGCATCAGGCTTGCGCCCATTGTGCAATATTCCCCACTGCTGCCTCCCGTAGGAGTCTGGACCGTGTCTCAGTTCCAGTGTGGCTGGTCATCCTCTCAGACCAGCTAGGGATCGTCGCCTAGGTGAGCCATTACCCCACCTACTAGCTAATCCCATCTGGGCACATCTGATGGCATGAGGCCCGAAGGTCCCCCACTTTGGTCCGAAGACGTTATGCGGTATTAGCTACCGTTTCCAGTAGTTATCCCCCTCCATCAGGCAGTTTCCCAGACATTACTCACCCGTCCGCCGCTCGTCACCCAGGAGCAAGCTCCCTGGTGTTACCGCTCGACTGCAGGTAGG

> Enterobacter ludwigii SLT7

CTGTCAACTTAGGCGGCTGGCTCCATAAAGGTTACCTCACCGACTTCGGGTGTTCAAACTCTCGTGGTGTGACGGGCGGTGTGTACAAGGCCCGGGAACGTATTCACCGCGGCATGCTGATCCGCGATTACTAGCGATTCCGGCTTCATGCAGGCGAGTTGCAGCCTGCAATCCGAACTGAGAATGGCTTTATGGGATTCGCTTACCTTCGCAGGTTTGCAGCCCTTTGTACCATCCATTGTAGCACGTGTGTAGCCCAGGTCATAAGGGGCATGATGATTTGACGTCATCCCCACCTTCCTCCGGTTTGTCACCGGCAGTCACCTTAGAGTGCCCAACTGAATGCTGGCAACTAAGATCAAGGGTTGCGCTCGTTGCGGGACTTAACCCAACATCTCACGACACGAGCTGACGACAACCATGCACCACCTGTCACTCTGTCCCCCGAAGGGGAAAGCCCTATCTCTAGGGTTGTCAGAGGATGTCAAGACCTGGTAAGGTTCTTCGCGTTGCTTCGAATTAAACCACATGCTCCACCGCTTGTGCGGGCCCCCGTCAATTCCTTTGAGTTTCAGCCTTGCGGCCGTACTCCCCAGGCGGAGTGCTTAATGCGTTAGCTGCAGCACTAAAGGGCGGAAACCCTCTAACACTTAGCACTCATCGTTTACGGCGTGGACTACCAGGGTATCTAATCCTGTTTGCTCCCCACGCTTTCGCGCCTCAGTGTCAGTTACAGACCAGAAAGTCGCCTTCGCCACTGGTGTTCCTCCAAATCTCTACGCATTTCACCGCTACACTTGGAATTCCACTTTCCTCTTCTGCACTCAAGTTCCCCAGTTTCCAATGACCCTCCACGGTTGAGCCGTGGGCTTTCACATCAGACTTAAGGAACCACCTGCGCGCGCTTTACGCCCAATAATTCCGGACAACGCTTGCCACCTACGTATTACCGCGGCTGCTGGCACGTAGTTAGCCGTGGCTTTCTGGTTAGGTACCGTCAAGGTACCAGCAGTTACTCTGGTACTTGTTCTTCCCTAACAACAGAACTTTACGACCCGAAGGCCTTCTTCGTTCACGCGGCGTTGCTCCGTCAGACTTTCGTCCATTGCGGAAGATTCCCTACTGCTGCCTCCCGTAGGAGTCTGGGCCGTGTCTCAGTCCCAGTGTGGCCGATCACCCTCTCAGGTCGGCTACGCATCGTCGCCTTGGTGAGCCATTACCTCACCAACTAGCTAATGCGCCGCGGGCCCATCTATAAGTGACAGCGTAAACCGTCTTTCCATCTTCTCTCATGCGAGAAAAGAACGTATCCGGTATTAGCTCCGGTTTCCCGAAGTTATCCCAGTCTTATAGGCAGGTTGCCCACGTGTTACTCACCCGTCCGCCGCTAATCTCAGGGAGCAAGCTCCCGTCGATCGCTCGACTGCA

> Sporosarcina aquimarina SLT8

ATTCTGTCACTTCGGCGGCTGGCTCCCGTAAGGGTTACCCCACCGACTTCGGGTGTTCAAACTCTCGTGGTGTGACGGGCGGTGTGTACAAGACCCGGGAACGTATTCACCGTGGCATGCTGATCCACGATTACTAGCGATTCCGGCTTCATGCAGGCGAGTTGCAGCCTACAATCCGAACTGGGAACGGTTTTCTGGGATTGGCTCCCCCTCGCGGGTTTGCAGCCCTCTGTACCGTCCATTGTAGCACGTGTGTAGCCCAGGTCATAAGGGGCATGATGATTTGACGTCATCCCCACCTTCCTCCGGTTTGTCACCGGCAGTCACCTTAGAGTGCCCAACTAAATGATGGCAACTAAGATTAAGGGTTGCGCTCGTTGCGGGACTTAACCCAACATCTCACGACACGAGCTGACGACAACCATGCACCACCTGTCACCACTGTCCCCGAAGGGAAAGACATGTCTCCATGCCGGTCAGTGGGATGTCAAGACCTGGTAAGGTTCTTCGCGTTGCTTCGAATTAAACCACATGCTCCACCGCTTGTGCGGGTCCCCGTCAATTCCTTTGAGTTTCAGCCTTGCGGCCGTACTCCCCAGGCGGAGTGCTTAATGCGTTAGCTGCAGCACTAAGGGGCGGAAACCCCCTAACACTTAGCACTCATCGTTTACGGCGTGGACTACCAGGGTATCTAATCCTGTTTGCTCCCCACGCTTTCGCGCCTCAGCGTCAGTTACAGACCAGAAAGCCGCCTTCGCCACTGGTGTTCCTCCACATCTCTACGCATTTCACCGCTACACGTGGAATTCCGCTTTCCTCTTCTGTACTCAAGTCCCCCAGTTTCCAATGACCCTCCACGGTTGAGCCGTGGGCTTTCACATCAGACTTAAAGGACCGCCTGCGCGCGCTTTACGCCCAATAATTCCGGACAACGCTTGCCACCTACGTATTACCGCGGCTGCTGGCACGTAGTTAGCCGTGGCTTTCTGACGAGGTACCGTCAAGGTACGGGCAGTTACTCCCGTACGTGTTCTTCCCTCGCAACAGAGCTTTACGATCCGAAAACCTTCTTCACTCACGCGGCATTGCTCCATCAGACTTTCGTCCATTGTGGAAGATTCCCTACTGCTGCCTCCCGTAGGAGTCTGGGCCGTGTCTCAGTCCCAGTGTGGCCGATCACCCTCTCAGGTCGGCTACGCATCGTTGCCTTGGTAGGCCATTACCCCACCAACTAGCTAATGCGCCGCGGGCCCATCCTGCAGTGACAGCCGAAACCGTCTTTCAGAATTCCTTCATGCGAAGGAATTGATTATTCGGTATTAGCCCCGGTTTCCCGGAGTTATCCCCATCTGCAGGGCAGGTTGCCCACGTGTTACTCACCCGTCCGCCGCTAATCTCAAGGAGCAAGCTCCTATCGATCGCTCGACTGCAGTATAGC

>Arthrobacter sp. SLT9

TTCGACGGCTCCCTCCCACAAGGGGTTAGGCCCCGGCTTCGGGTGTTACCAACTTTCGTGACTTGACGGGCGGTGTGTACAAGGCCCGGGAACGTATTCACCGCAGCGTTGCTGATCTGCGATTACTAGCGACTCCGACTTCATGGGGTCGAGTTGCAGACCCCAATCCGAACTGAGACCGGCTTTTTGGGATTAGCTCCACCTCACAGTATCGCAACCCATTGTACCGGCCATTGTAGCATGCGTGAAGCCCAAGACATAAGGGGCATGATGATTTGACGTCGTCCCCACCTTCCTCCGAGTTGACCCCGGCAGTCTCCCATGAGTCCCCGCCATTACGCGCTGGCAACATGGAACGAGGGTTGCGCTCGTTGCGGGACTTAACCCAACATCTCACGACACGAGCTGACGACAACCATGCACCACCTGTGAACCGGCCCCGAAGGGAAGGACTGTTTCCAGCCCGGTCCGGCCCATGTCAAGCCTTGGTAAGGTTCTTCGCGTTGCATCGAATTAATCCGCATGCTCCGCCGCTTGTGCGGGCCCCCGTCAATTCCTTTGAGTTTTAGCCTTGCGGCCGTACTCCCCAGGCGGGGCACTTAATGCGTTAGCTACGGCGCGGAAAACGTGGAATGTCCCCCACACCTAGTGCCCAACGTTTACGGCATGGACTACCAGGGTATCTAATCCTGTTCGCTCCCCATGCTTTCGCTCCTCAGCGTCAGTTACAGCCCAGAGACCTGCCTTCGCCATCGGTGTTCCTCCTGATATCTGCGCATTTCACCGCTACACCAGGAATTCCAGTCTCCCCTACTGCACTCTAGTCTGCCCGTACCCACTGCAGACCCGGGGTTGAGCCCCGGGCTTTCACAGCAGACGCGACAAACCGCCTACGAGCTCTTTACGCCCAATAATTCCGGACAACGCTTGCGCCCTACGTATTACCGCGGCTGCTGGCACGTAGTTAGCCGGCGCTTCTTCTGCAGGTACCGTCACTTTCGCTTCTTCCCTGCTGAAAGAGGTTTACAACCCGAAGGCATTCATCCCTCACGCGGCGTCGCTGCATCAGGCTTGCGCCCATTGTGCAATATTCCCCACTGCTGCCTCCCGTAGGAGTCTGGGCCGTGTCTCAGTCCCAGTGTGGCCGGTCACCCTCTCAGGCCGGCTACCCGTCGTCGCCTTGGTAGGCCATTACCCCACCAACAAGCTGATAGGCCGCGAGTCCATCCAAGACCAAAAAATCTTTCCACCCACCACCATGCGGTGACAGGTCATATCCGGTATTAGACCCGGTTTCCCAGGCTTATCCCAGAGTCAAGGGCAGGTTACTCACGTGTTACTCACCCGTTCGCCACTAATCCCCGGTGCAAGCACCGGATCATCGTCGACTGCAGGTAG

> Pseudomonas chlororaphis SLT10

TGCAAGTCGAGCGGATGACGGGAGCTTGCTCCCTAATTCAGCGGCGGACGGGTGAGTAATGCCTAGGAATCTGCCTGGTAGTGGGGGACAACGTCTCGAAAGGGACGCTAATACCGCATACGTCCTACGGGAGAAAGCAGGGGACCTTCGGGCCTTGCGCTATCAGATGAGCCTAGGTCGGATTAGCTAGTTGGTGAGGTAATGGCTCACCAAGGCGACGATCCGTAACTGGTCTGAGAGGATGATCAGTCACACTGGAACTGAGACACGGTCCAGACTCCTACGGGAGGCAGCAGTGGGGAATATTGGACAATGGGCGAAAGCCTGATCCAGCCATGCCGCGTGTGTGAAGAAGGTCTTCGGATTGTAAAGCACTTTAAGTTGGGAGGAAGGGCATTAACCTAATACGTTAGTGTTTTGACGTTACCGACAGAATAAGCACCGGCTAACTCTGTGCCAGCAGCCGCGGTAATACAGAGGGTGCAAGCGTTAATCGGAATTACTGGGCGTAAAGCGCGCGTAGGTGGTTTGTTAAGTTGGATGTGAAAGCCCCGGGCTCAACCTGGGAACTGCATTCAAAACTGACAAGCTAGAGTATGGTAGAGGGTGGTGGAATTTCCTGTGTAGCGGTGAAATGCGTAGATATAGGAAGGAACACCAGTGGCGAAGGCGACCACCTGGACTGATACTGACACTGAGGTGCGAAAGCGTGGGGAGCAAACAGGATTAGATACCCTGGTAGTCCACGCCGTAAACGATGTCAACTAGCCGTTGGGAGCCTTGAGCTCTTAGTGGCGCAGCTAACGCATTAAGTTGACCGCCTGGGGAGTACGGCCGCAAGGTTAAAACTCAAATGAATTGACGGGGGCCCGCACAAGCGGTGGAGCATGTGGTTTAATTCGAAGCAACGCGAAGAACCTTACCAGGCCTTGACATCCAATGAACTTTCCAGAGATGGATTGGTGCCTTCGGGAACATTGAGACAGGTGCTGCATGGCTGTCGTCAGCTCGTGTCGTGAGATGTTGGGTTAAGTCCCGTAACGAGCGCAACCCTTGTCCTTAGTTACCAGCACGTAATGGTGGGCACTCTAAGGAGACTGCCGGTGACAAACCGGAGGAAGGTGGGGATGACGTCAAGTCATCATGGCCCTTACGGCCTGGGCTACACACGTGCTACAATGGTCGGTACAGAGGGTTGCCAAGCCGCGAGGTGGAGCTAATCCCATAAAACCGATCGTAGTCCGGATCGCAGTCTGCAACTCGACTGCGTGAAGTCGGAATCGCTAGTAATCGCGAATCAGAATGTCGCGGTGAATACGTTCCCGGGCCTTGTACACACCGCCCGTCACACCATGGGAGTGGGTTGCACCAGAAGTAGCTAGTCTAACCTTCGGGAGGAC

> Bacillus wiedmannii SLT11

TTAGGCGGCTGGCTCCATAAAGGTTACCCCACCGACTTCGGGTGTTACAAACTCTCGTGGTGTGACGGGCGGTGTGTACAAGGCCCGGGAACGTATTCACCGCGGCATGCTGATCCGCGATTACTAGCGATTCCAGCTTCATGTAGGCGAGTTGCAGCCTACAATCCGAACTGAGAACGGTTTTATGAGATTAGCTCCACCTCGCGGTCTTGCAGCTCTTTGTACCGTCCATTGTAGCACGTGTGTAGCCCAGGTCATAAGGGGCATGATGATTTGACGTCATCCCCACCTTCCTCCGGTTTGTCACCGGCAGTCACCTTAGAGTGCCCAACTTAATGATGGCAACTAAGATCAAGGGTTGCGCTCGTTGCGGGACTTAACCCAACATCTCACGACACGAGCTGACGACAACCATGCACCACCTGTCACTCTGCTCCCGAAGGAGAAGCCCTATCTCTAGGGTTTTCAGAGGATGTCAAGACCTGGTAAGGTTCTTCGCGTTGCTTCGAATTAAACCACATGCTCCACCGCTTGTGCGGGCCCCCGTCAATTCCTTTGAGTTTCAGCCTTGCGGCCGTACTCCCCAGGCGGAGTGCTTAATGCGTTAACTTCAGCACTAAAGGGCGGAAACCCTCTAACACTTAGCACTCATCGTTTACGGCGTGGACTACCAGGGTATCTAATCCTGTTTGCTCCCCACGCTTTCGCGCCTCAGTGTCAGTTACAGACCAGAAAGTCGCCTTCGCCACTGGTGTTCCTCCATATCTCTACGCATTTCACCGCTACACATGGAATTCCACTTTCCTCTTCTGCACTCAAGTCTCCCAGTTTCCAATGACCCTCCACGGTTGAGCCGTGGGCTTTCACATCAGACTTAAGAAACCACCTGCGCGCGCTTTACGCCCAATAATTCCGGATAACGCTTGCCACCTACGTATTACCGCGGCTGCTGGCACGTAGTTAGCCGTGGCTTTCTGGTTAGGTACCGTCAAGGTGCCAGCTTATTCAACTAGCACTTGTTCTTCCCTAACAACAGAGTTTTACGACCCGAAAGCCTTCATCACTCACGCGGCGTTGCTCCGTCAGACTTTCGTCCATTGCGGAAGATTCCCTACTGCTGCCTCCCGTAGGAGTCTGGGCCGTGTCTCAGTCCCAGTGTGGCCGATCACCCTCTCAGGTCGGCTACGCATCGTTGCCTTGGTGAGCCGTTACCTCACCAACTAGCTAATGCGACGCGGGTCCATCCATAAGTGACAGCCGAAGCCGCCTTTCAATTTCGAACCATGCAGTTCAAAATGTTATCCGGTATTAGCCCCGGTTTCCCGGAGTTATCCCAGTCTTATGGGCAGGTTACCCACGTGTTACTCACCCGTCCGCCGCTAACTTCATAAGAGCAAGCTCTCAATCCATTCGCTCGACTTGC

>Ensifer adhaerens SLT12

CCCCGCAAGGGGAGCGGCAGACGGGTGAGTAACGCGTGGGAATCTACCCTTTTCTACGGAATAACGCATGGAAACGTGTGCTAATACCGTATGAGCCCTTCGGGGGAAAGATTTATCGGGAAAGGATGAGCCCGCGTTGGATTAGCTAGTTGGTGGGGTAAAGGCCTACCAAGGCGACGATCCATAGCTGGTCTGAGAGGATGATCAGCCACATTGGGACTGAGACACGGCCCAAACTCCTACGGGAGGCAGCAGTGGGGAATATTGGACAATGGGCGCAAGCCTGATCCAGCCATGCCGCGTGAGTGATGAAGGCCCTAGGGTTGTAAAGCTCTTTCACCGGTGAAGATAATGACGGTAACCGGAGAAGAAGCCCCGGCTAACTTCGTGCCAGCAGCCGCGGTAATACGAAGGGGGCTAGCGTTGTTCGGAATTACTGGGCGTAAAGCGCACGTAGGCGGACATTTAAGTCAGGGGTGAAATCCCAGAGCTCAACTCTGGAACTGCCTTTGATACTGGGTGTCTAGAGTATGGAAGAGGTGAGTGGAATTCCGAGTGTAGAGGTGAAATTCGTAGATATTCGGAGGAACACCAGTGGCGAAGGCGGCTCACTGGTCCATTACTGACGCTGAGGTGCGAAAGCGTGGGGAGCAAACAGGATTAGATACCCTGGTAGTCCACGCCGTAAACGATGAATGTTAGCCGTCGGGCAGTTTACTGTTCGGTGGCGCAGCTAACGCATTAAACATTCCGCCTGGGGAGTACGGTCGCAAGATTAAAACTCAAAGGAATTGACGGGGGCCCGCACAAGCGGTGGAGCATGTGGTTTAATTCGAAGCAACGCGCAGAACCTTACCAGCCCTTGACATCCCGATCGCGGATTACAGAGATGTAGTCCTTCAGTTCGGCTGGATCGGAGACAGGTGCTGCATGGCTGTCGTCAGCTCGTGTCGTGAGATGTTGGGTTAAGTCCCGCAACGAGCGCAACCCTCGCCCTTAGTTGCCAGCATTAAGTTGGGCACTCTAAGGGGACTGCCGGTGATAAGCCGAGAGGAAGGTGGGGATGACGTCAAGTCCTCATGGCCCTTACGGGCTGGGCTACACACGTGCTACAATGGTGGTGACAGTGGGCAGCGAGACCGCGAGGTCGAGCTAATCTCCAAAAGCCATCTCAGTTCGGATTGCACTCTGCAACTCGAGTGCATGAAGTTGGAATCGCTAGTAATCGCAGATCAGCATGCTGCGGTGAATACGTTCCCGGGCCTTGTACACACCGCCCGTCACACCATGGGAGTTGGTTCTACCCGAAGGTAGTGCGCTAACCGCAAGGAGGCAGC

>Pseudarthrobacter sp. SLT13

TGCAAGTCGAACGATGATCCCAGCTTGCTGGGGGATTAGTGGCGAACGGGTGAGTAACACGTGAGTAACCTGCCCTTAACTCTGGGATAAGCCTGGGAAACTGGGTCTAATACCGGATATGACTCCTCATCGCATGGTGGGGGGTGGAAAGCTTTATTGTGGTTTTGGATGGACTCGCGGCCTATCAGCTTGTTGGTGAGGTAATGGCTCACCAAGGCGACGACGGGTAGCCGGCCTGAGAGGGTGACCGGCCACACTGGGACTGAGACACGGCCCAGACTCCTACGGGAGGCAGCAGTGGGGAATATTGCACAATGGGCGAAAGCCTGATGCAGCGACGCCGCGTGAGGGATGACGGCCTTCGGGTTGTAAACCTCTTTCAGTAGGGAAGAAGCGAAAGTGACGGTACCTGCAGAAGAAGCGCCGGCTAACTACGTGCCAGCAGCCGCGGTAATACGTAGGGCGCAAGCGTTATCCGGAATTATTGGGCGTAAAGAGCTCGTAGGCGGTTTGTCGCGTCTGCCGTGAAAGTCCGGGGCTCAACTCCGGATCTGCGGTGGGTACGGGCAGACTAGAGTGATGTAGGGGAGACTGGAATTCCTGGTGTAGCGGTGAAATGCGCAGATATCAGGAGGAACACCGATGGCGAAGGCAGGTCTCTGGGCATTAACTGACGCTGAGGAGCGAAAGCATGGGGAGCGAACAGGATTAGATACCCTGGTAGTCCATGCCGTAAACGTTGGGCACTAGGTGTGGGGGACATTCCACGTTTTCCGCGCCGTAGCTAACGCATTAAGTGCCCCGCCTGGGGAGTACGGCCGCAAGGCTAAAACTCAAAGGAATTGACGGGGGCCCGCACAAGCGGCGGAGCATGCGGATTAATTCGATGCAACGCGAAGAACCTTACCAAGGCTTGACATGAACCGGAAACACCTGGAAACAGGTGCCCCGCTTGCGGTCGGTTTACAGGTGGTGCATGGTTGTCGTCAGCTCGTGTCGTGAGATGTTGGGTTAAGTCCCGCAACGAGCGCAACCCTCGTTCTATGTTGCCAGCGCGTTATGGCGGGGACTCATAGGAGACTGCCGGGGTCAACTCGGAGGAAGGTGGGGACGACGTCAAATCATCATGCCCCTTATGTCTTGGGCTTCACGCATGCTACAATGGCCGGTACAAAGGGTTGCGATACTGTGAGGTGGAGCTAATCCCAAAAAGCCGGTCTCAGTTCGGATTGGGGTCTGCAACTCGACCCCATGAAGTCGGAGTCGCTAGTAATCGCAGATCAGCAACGCTGCGGTGAATACGTTCCCGGGCCTTGTACACACCGCCCGTCAAGTCACGAAAGTTGGTAACACCCGAAGCCGGTGGCCTAACCCCTTGTGGGAGGGAGCTGTCGAA

>Flavobacterium edaphi SLT14

TGCAGTCGAGGGGTAGAAGTCTTCGGACTTTGAGACCGGCGCACGGGTGCGTAACGCGTATGCAATCTGCCTTGTACAAAGGGATAGCCCAGAGAAATTTGGATTAATACCTTATAGTATATAGATGTGGCATCACATTTATATTAAAGATTTATCGGTACAAGATGAGCATGCGTCCCATTAGCTAGTTGGTATGGTAACGGCATACCAAGGCAATGATGGGTAGGGGTCCTGAGAGGGAGATCCCCCACACTGGTACTGAGACACGGACCAGACTCCTACGGGAGGCAGCAGTGAGGAATATTGGTCAATGGGCGCAAGCCTGAACCAGCCATGCCGCGTGCAGGATGAAGCATCTATGGTGTGTAAACTGCTTTTGTACGGGAAGAAACAACTCTTCGTGAAGAGTCTTGACGGTACCGTAAGAATAAGGATCGGCTAACTCCGTGCCAGCAGCCGCGGTAATACGGAGGATCCAAGCGTTATCCGGAATCATTGGGTTTAAAGGGTCCGTAGGCGGTTTTATAAGTCAGTGGTGAAATCCGGCAGCTTAACTGTCGAACTGCCATTGATACTGTAGAACTTGAATTATTGTGAAGTAACTAGAATATGTAGTGTAGCGGTGAAATGCTTAGATATTACATGGAATACCAATTGCGAAGGCAGGTTACTAACAATACATTGACGCTGATGGACGAAAGCGTGGGGAGCGAACAGGATTAGATACCCTGGTAGTCCACGCCGTAAACGATGGATACTAGCTGTTTGGTAGCAATACTGAGTGGCTAAGCGAAAGTGATAAGTATCCCACCTGGGGAGTACGAACGCAAGTTTGAAACTCAAAGGAATTGACGGGGGCCCGCACAAGCGGTGGAGCATGTGGTTTAATTCGATGATACGCGAGGAACCTTACCAGGGCTTAAATGTAGTGGGACAGGCTTGGAAACAGGCCCTTCTTCGGACTCATTACAAGGTGCTGCATGGTTGTCGTCAGCTCGTGCCGTGAGGTGTCAGGTTAAGTCCTATAACGAGCGCAACCCCTGTTGTTAGTTGCCAGCGAGTAATGTCGGGAACTCTAACAAGACTGCCGGTGCAAACCGTGAGGAAGGTGGGGATGACGTCAAATCATCACGGCCCTTACGTCCTGGGCTACACACGTGCTACAATGGCCGGTACAGAGAGCAGCCACCTCGCGAGGGGGAGCGAATCTATAAAACCGGTCACAGTTCGGATTGGAGTCTGCAACCCGACTCCATGAAGCTGGAATCGCTAGTAATCGGATATCAGCCATGATCCGGTGAATACGTTCCCGGGCCTTGTACACACCGCCCGTCAAGCCATGGAAGCTGGGGGTACCTGAAGTCGGTGACCGCAAGGAGCTGCCT

>Oerskovia enterophila SLT15

TCCCTCCACAAGGGTTGGGCCACCGGCTTCGGGTGTTACCGACTTTCGTGACTTGACGGGCGGTGTGTACAAGGCCCGGGAACGTATTCACCGCAGCGTTGCTGATCTGCGATTACTAGCGACTCCGACTTCATGGGGTCGAGTTGCAGACCCCAATCCGAACTGAGACCGGCTTTTTGGGATTCGCTCCACCTCGCGGTATCGCAGCCCTTTGTACCGGCCATTGTAGCATGCGTGAAGCCCAAGACATAAGGGGCATGATGATTTGACGTCATCCCCACCTTCCTCCGAGTTGACCCCGGCAGTCTCTTATGAGTCCCCACCATGACGTGCTGGCAACATAAGACGAGGGTTGCGCTCGTTGCGGGACTTAACCCAACATCTCACGACACGAGCTGACGACAACCATGCACCACCTGTACACCGACCTTGCGGGGCAACCATCTCTGGAAGTTTCCGGTGTATGTCAAGCCTTGGTAAGGTTCTTCGCGTTGCATCGAATTAATCCGCATGCTCCGCCGCTTGTGCGGGCCCCCGTCAATTCCTTTGAGTTTTAGCCTTGCGGCCGTACTCCCCAGGCGGGGCACTTAATGCGTTTGCTGCGGCACGGAACTCGTGGAATGAGCCCCACACCTAGTGCCCAACGTTTACGGCATGGACTACCAGGGTATCTAATCCTGTTCGCTCCCCATGCTTTCGCTCCTCAGCGTCAGTTGCGGCCCAGTGACCTGCCTTCGCCATCGGTGTTCCTCCTGATATCTGCGCATTCCACCGCTACACCAGGAATTCCAGTCACCCCTACCGCACTCTAGTCTGCCCGTACCCGATGCAAGCTCAAGGTTGAGCCTTGAGTTTTCACACCAGACGCGACAAACCGCCTACGAGCTCTTTACGCCCAATAATTCCGGACAACGCTTGCGCCCTACGTATTACCGCGGCTGCTGGCACGTAGTTAGCCGGCGCTTCTTCTGCAGGTACCGTCACTTGCGCTTCTTCCCTGCTGAAAGAGGTTTACAACCCGAAGGCCTTCATCCCTCACGCGGCGTCGCTGCATCAGGCTTGCGCCCATTGTGCAATATTCCCCACTGCTGCCTCCCGTAGGAGTCTGGGCCGTGTCTCAGTCCCAGTGTGGCCGGTCGCCCTCTCAGGCCGGCTACCCGTCGTCGCCTTGGTAGGCCATTACCCCACCAACAAGCTGATAGGCCGCGAGTCCATCCCAGACCGATAAATCTTTCCAGACACTCCCCATGCAGGGGCATCTCATATCCAGTATTAGACCTCGTTTCCAAGGCTTATCCCAGAGTCTGGGGCAGGTTACTCACGTGTTACTCACCCGTTCGCCACTGATCCGACCAGCAAGCTGGTCATCACCGTTCGACTGCA

>Microbacterium sp SLT16

ACGGCTCCCTCCACAAGGGTTGGGCCACCGGCTTCAGGTGTTACCGACTTTCATGACTTGACGGGCGGTGTGTACAAGACCCGGGAACGTATTCACCGCAGCGTTGCTGATCTGCGATTACTAGCGACTCCGACTTCATGAGGTCGAGTTGCAGACCTCAATCCGAACTGGGACCGGCTTTTTGGGATTCGCTCCACCTCACGGTATTGCAGCCCTTTGTACCGGCCATTGTAGCATGCGTGAAGCCCAAGACATAAGGGGCATGATGATTTGACGTCATCCCCACCTTCCTCCGAGTTGACCCCGGCAGTATCCCATGAGTTCCCACCATTACGTGCTGGCAACATAGAACGAGGGTTGCGCTCGTTGCGGGACTTAACCCAACATCTCACGACACGAGCTGACGACAACCATGCACCACCTGTTCACGAGTGTCCAAAGAGTCCCCTATTTCTAGAGTGTTCTCGTGTATGTCAAGCCTTGGTAAGGTTCTTCGCGTTGCATCGAATTAATCCGCATGCTCCGCCGCTTGTGCGGGTCCCCGTCAATTCCTTTGAGTTTTAGCCTTGCGGCCGTACTCCCCAGGCGGGGAACTTAATGCGTTAGCTGCGTCACGGAATCCGTGGAAAGGACCCCACAACTAGTTCCCAACGTTTACGGGGTGGACTACCAGGGTATCTAAGCCTGTTTGCTCCCCACCCTTTCGCTCCTCAGCGTCAGTTACGGCCCAGAGATCTGCCTTCGCCATCGGTGTTCCTCCTGATATCTGCGCATTCCACCGCTACACCAGGAATTCCAATCTCCCCTACCGCACTCTAGTCTGCCCGTACCCACTGCAGGCCCGAGGTTGAGCCTCGGGATTTCACAGCAGACGCGACAAACCGCCTACGAGCTCTTTACGCCCAATAATTCCGGATAACGCTTGCGCCCTACGTATTACCGCGGCTGCTGGCACGTAGTTAGCCGGCGCTTTTTCTGCAAGTACCGTCACTTTCGCTTCTTCCTTGCTAAAAGAGGTTTACAACCCGAAGGCCGTCATCCCTCACGCGGCGTTGCTGCATCAGGCTTCCGCCCATTGTGCAATATTCCCCACTGCTGCCTCCCGTAGGAGTCTGGGCCGTGTCTCAGTCCCAGTGTGGCCGGTCACCCTCTCAGGCCGGCTACCCGTCGACGCCTTGGTGAGCCATTACCTCACCAACAAGCTGATAGGCCGCGAGCCCATCCCAGACCGAAAAATCTTTCCAACCCCCACCATGCGGTAGAAGCTCATATCCAGTATTAGACGCCGTTTCCAGCGCTTATCCCAGAGTCCAGGGCAGGTTGCTCACGTGTTACTCACCCGTTCGCCACTGATCCACCAAGCAAGCTTGGCTTCACCGTTCGACTGCA

>Arthrobacter sp. SLT17

TGCAGTCGAACGATGATCCGGTGCTTGCACCGGGGATTAGTGGCGAACGGGTGAGTAACACGTGAGTAACCTGCCCTTGACTCTGGGATAAGCCTGGGAAACCGGGTCTAATACCGGATATGACCTGTCACCGCATGGTGGTGGGTGGAAAGATTTTTTGGTCTTGGATGGACTCGCGGCCTATCAGCTTGTTGGTGGGGTAATGGCCTACCAAGGCGACGACGGGTAGCCGGCCTGAGAGGGTGACCGGCCACACTGGGACTGAGACACGGCCCAGACTCCTACGGGAGGCAGCAGTGGGGAATATTGCACAATGGGCGCAAGCCTGATGCAGCGACGCCGCGTGAGGGATGAATGCCTTCGGGTTGTAAACCTCTTTCAGCAGGGAAGAAGCGAAAGTGACGGTACCTGCAGAAGAAGCGCCGGCTAACTACGTGCCAGCAGCCGCGGTAATACGTAGGGCGCAAGCGTTGTCCGGAATTATTGGGCGTAAAGAGCTCGTAGGCGGTTTGTCGCGTCTGCTGTGAAAGCCCGGGGCTCAACCCCGGGTCTGCAGTGGGTACGGGCAGACTAGAGTGCAGTAGGGGAGACTGGAATTCCTGGTGTAGCGGTGAAATGCGCAGATATCAGGAGGAACACCGATGGCGAAGGCAGGTCTCTGGGCTGTAACTGACGCTGAGGAGCGAAAGCATGGGGAGCGAACAGGATTAGATACCCTGGTAGTCCATGCCGTAAACGTTGGGCACTAGGTGTGGGGGACATTCCACGTTTTCCGCGCCGTAGCTAACGCATTAAGTGCCCCGCCTGGGGAGTACGGCCGCAAGGCTAAAACTCAAAGGAATTGACGGGGGCCCGCACAAGCGGCGGAGCATGCGGATTAATTCGATGCAACGCGAAGAACCTTACCAAGGCTTGACATGGGCCGGACCGGGCTGGAAACAGTCCTTCCCTTCGGGGCCGGTTCACAGGTGGTGCATGGTTGTCGTCAGCTCGTGTCGTGAGATGTTGGGTTAAGTCCCGCAACGAGCGCAACCCTCGTTCCATGTTGCCAGCGCGTAATGGCGGGGACTCATGGGAGACTGCCGGGGTCAACTCGGAGGAAGGTGGGGACGACGTCAAATCATCATGCCCCTTATGTCTTGGGCTTCACGCATGCTACAATGGCCGGTACAATGGGTTGCGATACTGTGAGGTGGAGCTAATCCCAAAAAGCCGGTCTCAGTTCGGATTGGGGTCTGCAACTCGACCCCATGAAGTCGGAGTCGCTAGTAATCGCAGATCAGCAACGCTGCGGTGAATACGTTCCCGGGCCTTGTACACACCGCCCGTCAAGTCACGAAAGTTGGTAACACCCGAAGCCGGTGGCCTAACCCCTTGTGGGAGGGAGCCGTCGAA

>Sphingopyxis sp. SLT18

TGCAAGTCGAACGAACTCTTCGGAGTTAGTGGCGCACGGGTGCGTAACGCGTGGGAATCTGCCCTTGGGTACGGAATAACTCAGAGAAATTTGTGCTAATACCGTATAATGTCTTCGGACCAAAGATTTATCGCCCAAGGATGAGCCCGCGTAAGATTAGCTAGTTGGTGAGGTAAAAGCTCACCAAGGCGACGATCTTTAGCTGGTCTGAGAGGATGATCAGCCACACTGGGACTGAGACACGGCCCAGACTCCTACGGGAGGCAGCAGTGGGGAATATTGGACAATGGGCGAAAGCCTGATCCAGCAATGCCGCGTGAGTGATGAAGGCCTTAGGGTTGTAAAGCTCTTTTACCCGGGATGATAATGACAGTACCGGGAGAATAAGCTCCGGCTAACTCCGTGCCAGCAGCCGCGGTAATACGGAGGGAGCTAGCGTTGTTCGGAATTACTGGGCGTAAAGCGCGCGTAGGCGGTTTTTTAAGTCAGAGGTGAAAGCCCGGGGCTCAACCCCGGAATAGCCTTTGAAACTGGAAAACTTGAATCTTGGAGAGGTCAGTGGAATTCCGAGTGTAGAGGTGAAATTCGTAGATATTCGGAAGAACACCAGTGGCGAAGGCGACTGACTGGACAAGTATTGACGCTGAGGTGCGAAAGCGTGGGGAGCAAACAGGATTAGATACCCTGGTAGTCCACGCCGTAAACGATGATAACTATCTGTCCGGGCTCATAGAGCTTGGGTGGAGCAGCTAACGCATTAAGTTATCCGCCTGGGGAGTACGGTCGCAAGATTAAAACTCAAAGGAATTGACGGGGGCCTGCACAAGCGGTGGAGCATGTGGTTTAATTCGAAGCAACGCGCAGAACCTTACCAGCGTTTGACATCCTGATCGCGGATTAGAGAGATCTTTTCCTTCAGTTCGGCTGGATCAGTGACAGGTGCTGCATGGCTGTCGTCAGCTCGTGTCGTGAGATGTTGGGTTAAGTCCCGCAACGAGCGCAACCCTCATCCCTAGTTGCCATCATTTAGTTGGGCACTCTATGGAAACTGCCGGTGATAAGCCGGAGGAAGGTGGGGATGACGTCAAGTCCTCATGGCCCTTACACGCTGGGCTACACACGTGCTACAATGGCAACTACAGTGGGCAGCAACCTCGCGAGGGGTAGCTAATCTCCAAAAGTTGTCTCAGTTCGGATTGTTCTCTGCAACTCGAGAGCATGAAGGCGGAATCGCTAGTAATCGCGGATCAGCATGCCGCGGTGAATACGTTCCCAGGCCTTGTACACACCGCCCGTCACACCATGGGAGTTGGTTTCACCCGAAGGCAGTGCTCTAACCCGCAAGGGAGGAA

>Sphingopyxis sp. SLT19

TGCAAGTCGAACGAACTCTTCGGAGTTAGTGGCGCACGGGTGCGTAACGCGTGGGAATCTGCCCTTGGGTACGGAATAACTCAGAGAAATTTGTGCTAATACCGTATAATGTCTTCGGACCAAAGATTTATCGCCCAAGGATGAGCCCGCGTAAGATTAGCTAGTTGGTGAGGTAAAAGCTCACCAAGGCGACGATCTTTAGCTGGTCTGAGAGGATGATCAGCCACACTGGGACTGAGACACGGCCCAGACTCCTACGGGAGGCAGCAGTGGGGAATATTGGACAATGGGCGAAAGCCTGATCCAGCAATGCCGCGTGAGTGATGAAGGCCTTAGGGTTGTAAAGCTCTTTTACCCGGGATGATAATGACAGTACCGGGAGAATAAGCTCCGGCTAACTCCGTGCCAGCAGCCGCGGTAATACGGAGGGAGCTAGCGTTGTTCGGAATTACTGGGCGTAAAGCGCGCGTAGGCGGTTTTTTAAGTCAGAGGTGAAAGCCCGGGGCTCAACCCCGGAATAGCCTTTGAAACTGGAAAACTTGAATCTTGGAGAGGTCAGTGGAATTCCGAGTGTAGAGGTGAAATTCGTAGATATTCGGAAGAACACCAGTGGCGAAGGCGACTGACTGGACAAGTATTGACGCTGAGGTGCGAAAGCGTGGGGAGCAAACAGGATTAGATACCCTGGTAGTCCACGCCGTAAACGATGATAACTATCTGTCCGGGCTCATAGAGCTTGGGTGGAGCAGCTAACGCATTAAGTTATCCGCCTGGGGAGTACGGTCGCAAGATTAAAACTCAAAGGAATTGACGGGGGCCTGCACAAGCGGTGGAGCATGTGGTTTAATTCGAAGCAACGCGCAGAACCTTACCAGCGTTTGACATCCTGATCGCGGATTAGAGAGATCTTTTCCTTCAGTTCGGCTGGATCAGTGACAGGTGCTGCATGGCTGTCGTCAGCTCGTGTCGTGAGATGTTGGGTTAAGTCCCGCAACGAGCGCAACCCTCATCCCTAGTTGCCATCATTTAGTTGGGCACTCTATGGAAACTGCCGGTGATAAGCCGGAGGAAGGTGGGGATGACGTCAAGTCCTCATGGCCCTTACACGCTGGGCTACACACGTGCTACAATGGCAACTACAGTGGGCAGCAACCTCGCGAGGGGTAGCTAATCTCCAAAAGTTGTCTCAGTTCGGATTGTTCTCTGCAACTCGAGAGCATGAAGGCGGAATCGCTAGTAATCGCGGATCAGCATGCCGCGGTGAATACGTTCCCAGGCCTTGTACACACCGCCCGTCACACCATGGGAGTTGGTTTCACCCGAAGGCAGTGCTCTAACCCGCAAGGGAGGAA

> Microbacterium flavescens SLT20

TTCGACGGCTCCCTCCACAAGGGTTGGGCCACCGGCTTCAGGTGTTACCGACTTTCATGACTTGACGGGCGGTGTGTACAAGACCCGGGAACGTATTCACCGCAGCGTTGCTGATCTGCGATTACTAGCGACTCCGACTTCATGAGGTCGAGTTGCAGACCTCAATCCGAACTGGGACCGGCTTTTTGGGATTCGCTCCACCTCACGGTATTGCAGCCCTTTGTACCGGCCATTGTAGCATGCGTGAAGCCCAAGACATAAGGGGCATGATGATTTGACGTCATCCCCACCTTCCTCCGAGTTGACCCCGGCAGTATCCCATGAGTTCCCACCATTACGTGCTGGCAACATAGAACGAGGGTTGCGCTCGTTGCGGGACTTAACCCAACATCTCACGACACGAGCTGACGACAACCATGCACCACCTGTTCACGAGTGTCCAAAGAGTTCCACATTTCTGCGGCGTTCTCGTGTATGTCAAGCCTTGGTAAGGTTCTTCGCGTTGCATCGAATTAATCCGCATGCTCCGCCGCTTGTGCGGGTCCCCGTCAATTCCTTTGAGTTTTAGCCTTGCGGCCGTACTCCCCAGGCGGGGAACTTAATGCGTTAGCTGCGTCACGGAATCCGTGGAATGGACCCCACAACTAGTTCCCAACGTTTACGGGGTGGACTACCAGGGTATCTAAGCCTGTTTGCTCCCCACCCTTTCGCTCCTCAGCGTCAGTTACGGCCCAGAGATCTGCCTTCGCCATCGGTGTTCCTCCTGATATCTGCGCATTCCACCGCTACACCAGGAATTCCAATCTCCCCTACCGCACTCTAGTCTGCCCGTACCCACTGCAGACCCGAGGTTGAGCCTCGGGATTTCACAGCAGACGCGACAAACCGCCTACGAGCTCTTTACGCCCAATAATTCCGGATAACGCTTGCGCCCTACGTATTACCGCGGCTGCTGGCACGTAGTTAGCCGGCGCTTTTTCTGCAGGTACCGTCACTTTCGCTTCTTCCCTGCTAAAAGAGGTTTACAACCCGAAGGCCGTCGTCCCTCACGCGGCGTTGCTGCATCAGGCTTCCGCCCATTGTGCAATATTCCCCACTGCTGCCTCCCGTAGGAGTCTGGGCCGTGTCTCAGTCCCAGTGTGGCCGGTCACCCTCTCAGGCCGGCTACCCGTCGACGCCTTGGTGAGCCATTACCTCACCAACAAGCTGATAGGCCGCGAGCTCATCCCAGACCGAAAAATCTTTCCAACCACTCACCATGCGATGGTGGCTCATATCCGGTATTAGACGCCGTTTCCAGCGCTTATCCCAGAGTCAGGGGCAGATTGCTCACGTGTTACTCACCCGTTCGCCACTGATCCACCCAGCAAGCTGGGCTTCACCGTTCGACTTGCA

>Streptomyces phaeochromogenes SLT21

TGCAGTCGAACGATGAAGCCCTTCGGGGTGGATTAGTGGCGAACGGGTGAGTAACACGTGGGCAATCTGCCCTTCACTCTGGGACAAGCCCTGGAAACGGGGTCTAATACCGGATAACACCGGCTTCCGCATGGGAGCTGGTTGAAAGCTCCGGCGGTGAAGGATGAGCCCGCGGCCTATCAGCTTGTTGGTGGGGTAATGGCCTACCAAGGCGACGACGGGTAGCCGGCCTGAGAGGGCGACCGGCCACACTGGGACTGAGACACGGCCCAGACTCCTACGGGAGGCAGCAGTGGGGAATATTGCACAATGGGCGAAAGCCTGATGCAGCGACGCCGCGTGAGGGATGACGGCCTTCGGGTTGTAAACCTCTTTCAGCAGGGAAGAAGCGAAAGTGACGGTACCTGCAGAAGAAGCGCCGGCTAACTACGTGCCAGCAGCCGCGGTAATACGTAGGGCGCAAGCGTTGTCCGGAATTATTGGGCGTAAAGAGCTCGTAGGCGGCTTGTCACGTCGGGTGTGAAAGCCCGGGGCTTAACCCCGGGTCTGCATCCGATACGGGCAGGCTAGAGTGTGGTAGGGGAGATCGGAATTCCTGGTGTAGCGGTGAAATGCGCAGATATCAGGAGGAACACCGGTGGCGAAGGCGGATCTCTGGGCCATTACTGACGCTGAGGAGCGAAAGCGTGGGGAGCGAACAGGATTAGATACCCTGGTAGTCCACGCCGTAAACGTTGGGAACTAGGTGTTGGCGACATTCCACGTCGTCGGTGCCGCAGCTAACGCATTAAGTTCCCCGCCTGGGGAGTACGGCCGCAAGGCTAAAACTCAAAGGAATTGACGGGGGCCCGCACAAGCAGCGGAGCATGTGGCTTAATTCGACGCAACGCGAAGAACCTTACCAAGGCTTGACATATACCGGAAAGCATTAGAGATAGTGCCCCCCTTGTGGTCGGTATACAGGTGGTGCATGGCTGTCGTCAGCTCGTGTCGTGAGATGTTGGGTTAAGTCCCGCAACGAGCGCAACCCTTGTCCTGTGTTGCCAGCATGCCCTTCGGGGTGATGGGGACTCACAGGAGACCGCCGGGGTCAACTCGGAGGAAGGTGGGGACGACGTCAAGTCATCATGCCCCTTATGTCTTGGGCTGCACACGTGCTACAATGGCCGGTACAAAGAGCTGCGATGCCGCGAGGCGGAGCGAATCTCAAAAAGCCGGTCTCAGTTCGGATTGGGGTCTGCAACTCGACCCCATGAAGTCGGAGTTGCTAGTAATCGCAGATCAGCATTGCTGCGGTGAATACGTTCCCGGGCCTTGTACACACCGCCCGTCACGTCACGAAAGTCGGTAACACCCGAAGCCGGTGGCCCAACCCCTTGTGGGAGGGA

>Chitinophaga pinensis SLT22

GCAGTCGAGGGGCAGCGCAGTGTAGCAATACATGGGCGGCGACCGGCAAACGGGTGCGGAACACGTACGCAACCTTCCTTTAAGTGGGGAATAGCCCAGAGAAATTTGGATTAATACCCCGTAAGATGGTGGTCTGGCATCAGACAGCCATTAAAGCTGAGGCGCTTAAAGATGGGCGTGCGTCTGATTAGGTAGTTGGTGAGGTAATGGCTCACCAAGCCGACGATCAGTAACTGGCGTGAGAGCGCGACCAGTCACACGGGCACTGAGACACGGGCCCGACTCCTACGGGAGGCAGCAGTAAGGAATATTGGTCAATGGACGCAAGTCTGAACCAGCCATGCCGCGTGAAGGATGAAGGTCCTCTGGATTGTAAACTTCTTTTATGTGGGAAGAAACCACCTTTTTCTAAGGGTGTTGACGGTACCATAGGAATAAGCACCGGCTAACTCCGTGCCAGCAGCCGCGGTAATACGGAGGGTGCAAGCGTTATCCGGATTCACTGGGTTTAAAGGGTGCGTAGGCGGAATTGTAAGTCCGTGGTGAAATCTCCAAGCTTAACTTGGAAACTGCCATGGATACTATAGTTCTTGAATGTTGTGGAGGTTTGCGGAATATGTCATGTAGCGGTGAAATGCTTAGATATGACATAGAACACCGATTGCGAAGGCAGCAGGCTACACAAATATTGACGCTGAGGCACGAAAGCGTGGGGATCAAACAGGATTAGATACCCTGGTAGTCCACGCCCTAAACGATGATTACTCGACATTTGCGATATACAGTAAGTGTCTGAGCGAAAGCATTAAGTAATCCACCTGGGAAGTACGACCGCAAGGTTGAAACTCAAAGGAATTGACGGGGGTCCGCACAAGCGGTGGAGCATGTGGTTTAATTCGATGATACGCGAGGAACCTTACCTGGGCTAGAATGCTGGTGGACCGATCCTGAAAGGGATTTTTGTAGCAATACACCGCCAGTAAGGTGCTGCATGGCTGTCGTCAGCTCGTGCCGTGAGGTGTTGGGTTAAGTCCCGCAACGAGCGCAACCCCTATCATTAGTTGCCAACAGGTAAAGCTGGGAACTCTAATGAAACTGCCGTCGTAAGACGCGAGGAAGGAGGGGATGATGTCAAGTCATCATGGCCTTTATGCCCAGGGCTACACACGTGCTACAATGGTAGGAACAAAGGGCTGCTACCTGGTAACAGGCTGCTAATCTCAAAAATCCTATCTCAGTTCGGATTGAGGGCTGCAACTCGCCCTCATGAAGCTGGAATCGCTAGTAATCGTATATCAGCAATGATACGGTGAATACGTTCCCGGACCTTGTACACACCGCCCGTCAAGCCATGAAAGCCGGGGGGACCTGAAGTCGGCAACCGCAAGGAGCCGCC

>Agromyces sp. SLT23

TTCGACGGCTCCCTCCACAAGGGTTAGGCCACCGGCTTCGGGTGTTACCGACTTTCATGACTTGACGGGCGGTGTGTACAAGGCCCGGGAACGTATTCACCGCAGCGTTGCTGATCTGCGATTACTAGCGACTCCGACTTCATGAGGTCGAGTTGCAGACCTCAATCCGAACTGAGACCGGCTTTTTGGGATTCGCTCCGCCTTACGACATCGCAGCCCTTTGTACCGGCCATTGTAGCATGCGTGAAGCCCAAGACATAAGGGGCATGATGATTTGACGTCATCCCCACCTTCCTCCGAGTTGACCCCGGCAGTCTCACATGAGTTCCCACCATTACGTGCTGGCAACATGCGACGAGGGTTGCGCTCGTTGCGGGACTTAACCCAACATCTCACGACACGAGCTGACGACAACCATGCACCACCTGTAACCGAGTGTCCAAAGAGTTCCACATTTCTGCGGCGTTCTCGGTTATGTCAAGCCTTGGTAAGGTTCTTCGCGTTGCATCGAATTAATCCGCATGCTCCGCCGCTTGTGCGGGCCCCCGTCAATTCCTTTGAGTTTTAGCCTTGCGGCCGTACTCCCCAGGCGGGGCGCTTAATGCGTTAGCTACGACACGGAAACCGTGGAAAGGTCCCCACATCTAGCGCCCAACGTTTACGGCGTGGACTACCAGGGTATCTAATCCTGTTCGCTCCCCACGCTTTCGCTCCTCAGCGTCAGTTACGGCCCAGAGAACTGCCTTCGCCATCGGTGTTCCTCCTGATATCTGCGCATTCCACCGCTACACCAGGAATTCCATTCTCCCCTACCGCACTCCAGTCTGCCCGTACCCACTGCAGACCCGAGGTTGAGCCTCGGGATTTCACAGCAGACGCGACAAACCGCCTACGAGCTCTTTACGCCCAATAATTCCGGACAACGCTCGGACCCTACGTATTACCGCGGCTGCTGGCACGTAGTTAGCCGGTCCTTTTTCTGCAAGTACCGTCAAGAAGCAAGCTTCCCTTCTTCCTTACTAAAAGCGGTTTACAACCCGAAGGCCGTCATCCCGCACGCGGCGTTGCTGCATCAGGCTTGCGCCCATTGTGCAATATTCCCCACTGCTGCCTCCCGTAGGAGTCTGGGCCGTGTCTCAGTCCCAGTGTGGCCGGTCACCCTCTCAGGCCGGCTACCCGTCGACGCCTTGGTGAGCCATTACCTCACCAACAAGCTGATAGGCCGCGAGTCCATCCCAGACCGAAAAACTTTCCACCCCAAACCATGCGGTTCAAGGTCCTATCCGGTATTAGCTCCGATTTCTCGGGGTTATCCCAGAGTCCAGGGCAGGTTACTCACGTGTTACTCACCCGTTCGCCACTAATCCCCCCAGCAAGCTGGAGTTCATCGTTCGACTTGCA

>Sphingomonas sp. SLT24

TGCAGTCGAACGAACTCTTCGGAGTTAGTGGCGCACGGGTGCGTAACGCGTGGGAATCTGCCCTTGGGTACGGAATAACTCAGAGAAATTTGTGCTAATACCGTATAATGTCTTCGGACCAAAGATTTATCGCCCAAGGATGAGCCCGCGTAAGATTAGCTAGTTGGTGAGGTAAAAGCTCACCAAGGCGACGATCTTTAGCTGGTCTGAGAGGATGATCAGCCACACTGGGACTGAGACACGGCCCAGACTCCTACGGGAGGCAGCAGTGGGGAATATTGGACAATGGGCGAAAGCCTGATCCAGCAATGCCGCGTGAGTGATGAAGGCCTTAGGGTTGTAAAGCTCTTTTACCCGGGATGATAATGACAGTACCGGGAGAATAAGCTCCGGCTAACTCCGTGCCAGCAGCCGCGGTAATACGGAGGGAGCTAGCGTTGTTCGGAATTACTGGGCGTAAAGCGCGCGTAGGCGGTTTTTTAAGTCAGAGGTGAAAGCCCGGGGCTCAACCCCGGAATAGCCTTTGAAACTGGAAAACTTGAATCTTGGAGAGGTCAGTGGAATTCCGAGTGTAGAGGTGAAATTCGTAGATATTCGGAAGAACACCAGTGGCGAAGGCGACTGACTGGACAAGTATTGACGCTGAGGTGCGAAAGCGTGGGGAGCAAACAGGATTAGATACCCTGGTAGTCCACGCCGTAAACGATGATAACTATCTGTCCGGGCTCATAGAGCTTGGGTGGAGCAGCTAACGCATTAAGTTATCCGCCTGGGGAGTACGGTCGCAAGATTAAAACTCAAAGGAATTGACGGGGGCCTGCACAAGCGGTGGAGCATGTGGTTTAATTCGAAGCAACGCGCAGAACCTTACCAGCGTTTGACATCCTGATCGCGGATTAGAGAGATCTTTTCCTTCAGTTCGGCTGGATCAGTGACAGGTGCTGCATGGCTGTCGTCAGCTCGTGTCGTGAGATGTTGGGTTAAGTCCCGCAACGAGCGCAACCCTCATCCCTAGTTGCCATCATTTAGTTGGGCACTCTATGGAAACTGCCGGTGATAAGCCGGAGGAAGGTGGGGATGACGTCAAGTCCTCATGGCCCTTACACGCTGGGCTACACACGTGCTACAATGGCAACTACAGTGGGCAGCAACCTCGCGAGGGGTAGCTAATCTCCAAAAGTTGTCTCAGTTCGGATTGTTCTCTGCAACTCGAGAGCATGAAGGCGGAATCGCTAGTAATCGCGGATCAGCATGCCGCGGTGAATACGTTCCCAGGCCTTGTACACACCGCCCGTCACACCATGGGAGTTGGTTTCACCCGAAGGCAGTGCTCTAACCCGCAAGGGAGGAAGCTGACC

>Aminobacter aminovorans SLT25

GTGGTCCCTGCCTCCTTGCGGTTAGCACAGTGCCTTCGGGTAAAACCAACTCCCATGGTGTGACGGGCGGTGTGTACAAGGCCCGGGAACGTATTCACCGCAGCATGCTGATCTGCGATTACTAGCGATTCCAACTTCATGCACTCGAGTTGCAGAGTGCAATCCGAACTGAGATGGCTTTTGGAGATTAGCTCGACCTCGCGGTCTCGCTGCCCACTGTCACCACCATTGTAGCACGTGTGTAGCCCAGCCCGTAAGGGCCATGAGGACTTGACGTCATCTTCACCTTCCTCGCGGCTTATCACCGGCAGTCCCCTTAGAGTGCCCAACTTAATGATGGCAACTAAGGGCGAAGGTTGCGCTCGTTGCGGGACTTAACCCAACATCTCACGACACGAGCTGACGACAGCCATGCAGCACCTGTCACCGGTCCAGCCGAACTGAAGGGATCCATCTCTGGAAACCGCGACCGGGATGTCAAGGGCTGGTAAGGTTCTGCGCGTTGCTTCGAATTAAACCACATGCTCCACCGCTTGTGCGGGCCCCCGTCAATTCCTTTGAGTTTTAATCTTGCGACCGTACTCCCCAGGCGGAGAGCTTAATGCGTTAGCTGCGCCACCGACAAGTAAACTTGCCGACGGCTAGCTCTCATAGTTTACGGCGTGGACTACCAGGGTATCTAATCCTGTTTGCTCCCCACGCTTTCGCACCTCAGCGTCAGTACCGAGCCAGTGAGCCGCCTTCGCCACTGGTGTTCCTCCGAATATCTACGAATTTCACCTCTACACTCGGAATTCCACTCACCTCTCTCGGACTCGAGATTGCCAGTATTAAAGGCAGTTCCAGGGTTGAGCCCTGGGATTTCACCCCTAACTTAACAATCCGCCTACGTGCGCTTTACGCCCAGTAATTCCGAACAACGCTAGCCCCCTTCGTATTACCGCGGCTGCTGGCACGAAGTTAGCCGGGGCTTCTTCTCCGGTTACCGTCATTATCTTCACCGGTGAAAGAGCTTTACAACCCTAGGGCCTTCATCACTCACGCGGCATGGCTGGATCAGGCTTGCGCCCATTGTCCAATATTCCCCACTGCTGCCTCCCGTAGGAGTCTGGGCCGTGTCTCAGTCCCAGTGTGGCTGATCATCCTCTCAGACCAGCTATGGATCGTCGCCTTGGTAGGCCATTACCCCACCAACTAGCTAATCCAACGCGGGCTCATCCAACTCCGATAAATCTTTCTCCTATCGGACGTATACGGTATTAGCACAAGTTTCCCTGAGTTATTCCGTAGAGCTGGGTAGATTCCCACGCGTTACTCACCCGTCTGCCGCTCCCCTTGCGGGGCGCTCGACTGCA

>Lysinibacillus xylanilyticus SLT26

GCAAGTCGAGCGAACAGAGAAGGAGCTTGCTCCTTTGACGTTAGCGGCGGACGGGTGAGTAACACGTGGGCAACCTACCTTATAGTTTGGGATAACTCCGGGAAACCGGGGCTAATACCGAATAATCTATTTTACTTCATGGTGAAATACTGAAAGACGGTTTCGGCTGTCGCTATAAGATGGGCCCGCGGCGCATTAGCTAGTTGGTGAGGTAACGGCTCACCAAGGCGACGATGCGTAGCCGACCTGAGAGGGTGATCGGCCACACTGGGACTGAGACACGGCCCAGACTCCTACGGGAGGCAGCAGTAGGGAATCTTCCACAATGGGCGAAAGCCTGATGGAGCAACGCCGCGTGAGTGAAGAAGGTTTTCGGATCGTAAAACTCTGTTGTAAGGGAAGAACAAGTACAGTAGTAACTGGCTGTACCTTGACGGTACCTTATTAGAAAGCCACGGCTAACTACGTGCCAGCAGCCGCGGTAATACGTAGGTGGCAAGCGTTGTCCGGAATTATTGGGCGTAAAGCGCGCGCAGGCGGTCCTTTAAGTCTGATGTGAAAGCCCACGGCTCAACCGTGGAGGGTCATTGGAAACTGGGGGACTTGAGTGCAGAAGAGGAAAGTGGAATTCCAAGTGTAGCGGTGAAATGCGTAGAGATTTGGAGGAACACCAGTGGCGAAGGCGACTTTCTGGTCTGTAACTGACGCTGAGGCGCGAAAGCGTGGGGAGCAAACAGGATTAGATACCCTGGTAGTCCACGCCGTAAACGATGAGTGCTAAGTGTTAGGGGGTTTCCGCCCCTTAGTGCTGCAGCTAACGCATTAAGCACTCCGCCTGGGGAGTACGGTCGCAAGACTGAAACTCAAAGGAATTGACGGGGGCCCGCACAAGCGGTGGAGCATGTGGTTTAATTCGAAGCAACGCGAAGAACCTTACCAGGTCTTGACATCCCGTTGACCACTGTAGAGATATGGTTTTCCCTTCGGGGACAACGGTGACAGGTGGTGCATGGTTGTCGTCAGCTCGTGTCGTGAGATGTTGGGTTAAGTCCCGCAACGAGCGCAACCCTTGATCTTAGTTGCCATCATTTAGTTGGGCACTCTAAGGTGACTGCCGGTGACAAACCGGAGGAAGGTGGGGATGACGTCAAATCATCATGCCCCTTATGACCTGGGCTACACACGTGCTACAATGGACGATACAAACGGTTGCCAACTCGCGAGAGGGAGCTAATCCGATAAAGTCGTTCTCAGTTCGGATTGTAGGCTGCAACTCGCCTACATGAAGCCGGAATCGCTAGTAATCGCGGATCAGCATGCCGCGGTGAATACGTTCCCGGGCCTTGTACACACCGCCCGTCACACCACGAGAGTTTGTAACACCCGAAGTCGGTGGGGTAACCTTTTGGAGCCAGCCGCCGAA

>Pararhizobium herbae SLT27

GTGGTTAGCTGCCTCCTTGCGGTTAGCGCACTACCTTCGGGTAAAACCAACTCCCATGGTGTGACGGGCGGTGTGTACAAGGCCCGGGAACGTATTCACCGCAGCATGCTGATCTGCGATTACTAGCGATTCCAACTTCATGCACTCGAGTTGCAGAGTGCAATCCGAACTGAGATGGCTTTTGGAGATTAGCTCGACCTCGCGGTCTCGCTGCCCACTGTCACCACCATTGTAGCACGTGTGTAGCCCAGCCCGTAAGGGCCATGAGGACTTGACGTCATCCCCACCTTCCTCTCGGCTTATCACCGGCAGTCCCCTTAGAGTGCCCAACTGAATGCTGGCAACTAAGGGCGAGGGTTGCGCTCGTTGCGGGACTTAACCCAACATCTCACGACACGAGCTGACGACAGCCATGCAGCACCTGTCACCGATCCAGCCTAACTGAAGGACAATGTCTCCACTGTCCGCGATCGGGATGTCAAGAGCTGGTAAGGTTCTGCGCGTTGCTTCGAATTAAACCACATGCTCCACCGCTTGTGCGGGCCCCCGTCAATTCCTTTGAGTTTTAATCTTGCGACCGTACTCCCCAGGCGGAATGTTTAATGCGTTAGCTGCGCCACCGAACAGTAAACTGCCCGACGGCTAACATTCATCGTTTACGGCGTGGACTACCAGGGTATCTAATCCTGTTTGCTCCCCACGCTTTCGCACCTCAGCGTCAGTAATGGACCAGTGAGCCGCCTTCGCCACTGGTGTTCCTCCGAATATCTACGAATTTCACCTCTACACTCGGAATTCCACTCACCTCTTCCATACTCTAGACCGACAGTATCAAAGGCAGTTCCAGAGTTGAGCTCTGGGATTTCACCCCTGACTGATCGATCCGCCTACGTGCGCTTTACGCCCAGTAATTCCGAACAACGCTAGCCCCCTTCGTATTACCGCGGCTGCTGGCACGAAGTTAGCCGGGGCTTCTTCTCCGGATACCGTCATTATCTTCTCCGGTGAAAGTGCTTTACAACCCTAAGGCCTTCATCACACACGCGGCATGGCTGGATCAGGCTTGCGCCCATTGTCCAATATTCCCCACTGCTGCCTCCCGTAGGAGTTTGGGCCGTGTCTCAGTCCCAATGTGGCTGATCATCCTCTCAGACCAGCTATGGATCGTCGCCTTGGTAGGCCTTTACCCCACCAACTAGCTAATCCAACGCGGGCTCATCCATCTCCGATAAATCTTTCCCCAAAAGGGCGTATACGGTATTAGCACAAGTTTCCCTGAGTTATTCCGTAGAGATGGGTAGATTCCCACGCGTTACTCACCCGTCTGCCGCTCCCCTTGCGGGGCGCTCGACTGCA

>Chryseobacterium sp. SLT28

AGGCAGCTCCTGTTACGGTCACCGACTTCAGGTACCCCAGACTTCCATGGCTTGACGGGCGGTGTGTACAAGGCCCGGGAACGTATTCACCGCGCCATGGCTGATGCGCGATTACTAGCGATTCCAGCTTCATAGAGTCGAGTTGCAGACTCCAATCCGAACTGAGACCAGCTTTCGAGATTTGCATCACGTCACCGTGTAGCTGCCCTCTGTACTGGCCATTGTATTACGTGTGTGGCCCAAGGCGTAAGGGCCGTGATGATTTGACGTCATCCCCACCTTCCTCTCTACTTGCGTAGGCAGTCTCACTAGAGTCCCCAACTTAATGATGGCAACTAGTGACAGGGGTTGCGCTCGTTGCAGGACTTAACCTAACACCTCACGGCACGAGCTGACGACAACCATGCAGCACCTTGAAAAATGTCCGAAGAAAAGTCTATTTCTAAACCTGTCATTTCCCATTTAAGCCTTGGTAAGGTTCCTCGCGTATCATCGAATTAAACCACATAATCCACCGCTTGTGCGGGCCCCCGTCAATTCCTTTGAGTTTCATTCTTGCGAACGTACTCCCCAGGTGGCTAACTTATCACTTTCGCTTAGTCTCTGAATCCGAAAATCCAAAAACGAGTTAGCATCGTTTACGGCGTGGACTACCAGGGTATCTAATCCTGTTCGCTCCCCACGCTTTCGTCCATCAGCGTCAGTTGTTGCTTAGTAACCTGCCTTCGCAATTGGTGTTCTAAGTAATATCTATGCATTTCACCGCTACACTACTTATTCCAGCTACTTCAACAACACTCAAGACCTGCAGTATCAATGGCAGTTTCACAGTTAAGCTGTGAGATTTCACCACTGACTTACAGATCCGCCTACGGACCCTTTAAACCCAATAAATCCGGATAACGCTTGCACCCTCCGTATTACCGCGGCTGCTGGCACGGAGTTAGCCGGTGCTTATTCGTATAGTACCTTCAGCTACCCTCACGAGAGTAGGTTTATCCCTATACAAAAGAAGTTTACAACCCATAGGGCCGTCGTCCTTCACGCGGGATGGCTGGATCAGGCTCTCACCCATTGTCCAATATTCCTCACTGCTGCCTCCCGTAGGAGTCTGGTCCGTGTCTCAGTACCAGTGTGGGGGATCACCCTCTCAGGCCCCCTAAAGATCGTAGACTTGGTGAGCCGTTACCTCACCAACTATCTAATCTTGCGCGTGCCCATCTCTATCCACCGGAGTTTTCAATTTTAAATGATGCCATTCAAAATGTTATGGGGTATTAATCTTCCTTTCGAAAGGCTATCCCCCAGATAAAGGCAGGTTGCACACGTGTTCCGCACCCGTACGCCGCTCTCAAGATTCCGAAGAATCTCTACCGCTCGGCTGCA

>Pseudomonas putida SLT29

GTCCCCCCGAAGGTTAGACTAGCTACTTCTGGTGCAACCCACTCCCATGGTGTGACGGGCGGTGTGTACAAGGCCCGGGAACGTATTCACCGCGACATTCTGATTCGCGATTACTAGCGATTCCGACTTCACGCAGTCGAGTTGCAGACTGCGATCCGGACTACGATCGGTTTTGTGGGATTAGCTCCACCTCGCGGCTTGGCAACCCTCTGTACCGACCATTGTAGCACGTGTGTAGCCCAGGCCGTAAGGGCCATGATGACTTGACGTCATCCCCACCTTCCTCCGGTTTGTCACCGGCAGTCTCCTTAGAGTGCCCACCATTACGTGCTGGTAACTAAGGACAAGGGTTGCGCTCGTTACGGGACTTAACCCAACATCTCACGACACGAGCTGACGACAGCCATGCAGCACCTGTCTCAATGTTCCCGAAGGCACCAATCCATCTCTGGAAAGTTCATTGGATGTCAAGGCCTGGTAAGGTTCTTCGCGTTGCTTCGAATTAAACCACATGCTCCACCGCTTGTGCGGGCCCCCGTCAATTCATTTGAGTTTTAACCTTGCGGCCGTACTCCCCAGGCGGTCAACTTAATGCGTTAGCTGCGCCACTAAAGACTCAAGGTCCCCAACGGCTAGTTGACATCGTTTACGGCGTGGACTACCAGGGTATCTAATCCTGTTTGCTCCCCACGCTTTCGCACCTCAGTGTCAGTATCAGTCCAGGTGGTCGCCTTCGCCACTGGTGTTCCTTCCTATATCTACGCATTTCACCGCTACACAGGAAATTCCACCACCCTCTACCATACTCTAGCTTGTCAGTTTTGAATGCAGTTCCCAGGTTGAGCCCGGGGCTTTCACATCCAACTTAACAAACCACCTACGCGCGCTTTACGCCCAGTAATTCCGATTAACGCTTGCACCCTCTGTATTACCGCGGCTGCTGGCACAGAGTTAGCCGGTGCTTATTCTGTCGGTAACGTCAAAACACTAACGTATTAGGTTAATGCCCTTCCTCCCAACTTAAAGTGCTTTACAATCCGAAGACCTTCTTCACACACGCGGCATGGCTGGATCAGGCTTTCGCCCATTGTCCAATATTCCCCACTGCTGCCTCCCGTAGGAGTCTGGACCGTGTCTCAGTTCCAGTGTGACTGATCATCCTCTCAGACCAGTTACGGATCGTCGCCTTGGTGAGCCATTACCTCACCAACTAGCTAATCCGACCTAGGCTCATCTGATAGCGCAAGGCCCGAAGGTCCCCTGCTTTCTCCCGTAGGACGTATGCGGTATTAGCGTTCCTTTCGAAACGTTGTCCCCCACTACCAGGCAGATTCCTAGGCATTACTCACCCGTCCGCCGCTGAATCAGAGAGCAAGCTCTCTTCATCCGCTCGACTTGCA

>Streptomyces exfoliates SLT30

TGCAGTCGAACGATGAAGCCCTTCGGGGTGGATTAGTGGCGAACGGGTGAGTAACACGTGGGCAATCTGCCCTTCACTCTGGGACAAGCCCTGGAAACGGGGTCTAATACCGGATAACACCGGCTTCCGCATGGGGGCTGGTTAAAAGCTCCGGCGGTGAAGGATGAGCCCGCGGCCTATCAGCTTGTTGGTGGGGTAATGGCCTACCAAGGCGACGACGGGTAGCCGGCCTGAGAGGGCGACCGGCCACACTGGGACTGAGACACGGCCCAGACTCCTACGGGAGGCAGCAGTGGGGAATATTGCACAATGGGCGAAAGCCTGATGCAGCGACGCCGCGTGAGGGATGACGGCCTTCGGGTTGTAAACCTCTTTCAGCAGGGAAGAAGCGAAAGTGACGGTACCTGCAGAAGAAGCGCCGGCTAACTACGTGCCAGCAGCCGCGGTAATACGTAGGGCGCAAGCGTTGTCCGGAATTATTGGGCGTAAAGAGCTCGTAGGCGGCTTGTCACGTCGGGTGTGAAAGCCCGGGGCTTAACCCCGGGTCTGCATCCGATACGGGCAGGCTAGAGTGTGGTAGGGGAGATCGGAATTCCTGGTGTAGCGGTGAAATGCGCAGATATCAGGAGGAACACCGGTGGCGAAGGCGGATCTCTGGGCCATTACTGACGCTGAGGAGCGAAAGCGTGGGGAGCGAACAGGATTAGATACCCTGGTAGTCCACGCCGTAAACGTTGGGAACTAGGTGTTGGCGACATTCCACGTCGTCGGTGCCGCAGCTAACGCATTAAGTTCCCCGCCTGGGGAGTACGGCCGCAAGGCTAAAACTCAAAGGAATTGACGGGGGCCCGCACAAGCAGCGGAGCATGTGGCTTAATTCGACGCAACGCGAAGAACCTTACCAAGGCTTGACATATACCGGAAAGCATTAGAGATAGTGCCCCCCTTGTGGTCGGTATACAGGTGGTGCATGGCTGTCGTCAGCTCGTGTCGTGAGATGTTGGGTTAAGTCCCGCAACGAGCGCAACCCTTGTCCTGTGTTGCCAGCATGCCCTTCGGGGTGATGGGGACTCACAGGAGACCGCCGGGGTCAACTCGGAGGAAGGTGGGGACGACGTCAAGTCATCATGCCCCTTATGTCTTGGGCTGCACACGTGCTACAATGGCCGGTACAAAGAGCTGCGATGCCGCGAGGCGGAGCGAATCTCAAAAAGCCGGTCTCAGTTCGGATTGGGGTCTGCAACTCGACCCCATGAAGTCGGAGTTGCTAGTAATCGCAGATCAGCATTGCTGCGGTGAATACGTTCCCGGGCCTTGTACACACCGCCCGTCACGTCACGAAAGTCGGTAACACCCGAAGCCGGTGGCCCAACCCCTTGTGGGAGGGA

>Variovorax paradoxus SLT31

GGTATCGCCCTCCTTGCGGTTAAGCTAACTACTTCTGGCAGAACCCGCTCCCATGGTGTGACGGGCGGTGTGTACAAGACCCGGGAACGTATTCACCGTGACATTCTGATCCACGATTACTAGCGATTCCGACTTCACGCAGTCGAGTTGCAGACTGCGATCCGGACTACGACTGGTTTTATGGGATTAGCTCCCCCTCGCGGGTTGGCAACCCTTTGTACCAGCCATTGTATGACGTGTGTAGCCCCACCTATAAGGGCCATGAGGACTTGACGTCATCCCCACCTTCCTCCGGTTTGTCACCGGCAGTCTCATTAGAGTGCCCAACTGAATGTAGCAACTAATGACAAGGGTTGCGCTCGTTGCGGGACTTAACCCAACATCTCACGACACGAGCTGACGACAGCCATGCAGCACCTGTGTTACGGTTCTCTTTCGAGCACTAAGCCATCTCTGGCGAATTCCGTACATGTCAAAGGTGGGTAAGGTTTTTCGCGTTGCATCGAATTAAACCACATCATCCACCGCTTGTGCGGGTCCCCGTCAATTCCTTTGAGTTTCAACCTTGCGGCCGTACTCCCCAGGCGGTCAACTTCACGCGTTAGCTTCGTTACTGAGTCAGTGAAGACCCAACAACCAGTTGACATCGTTTAGGGCGTGGACTACCAGGGTATCTAATCCTGTTTGCTCCCCACGCTTTCGTGCATGAGCGTCAGTACAGGTCCAGGGGATTGCCTTCGCCATCGGTGTTCCTCCGCATATCTACGCATTTCACTGCTACACGCGGAATTCCATCCCCCTCTACCGTACTCTAGCTATGCAGTCACAGATGCAGTTCCCAGGTTGAGCCCGGGGATTTCACAACTGTCTTACATAACCGCCTGCGCACGCTTTACGCCCAGTAATTCCGATTAACGCTTGCACCCTACGTATTACCGCGGCTGCTGGCACGTAGTTAGCCGGTGCTTATTCTTACGGTACCGTCATTAGCCCTCTTTATTAGAAAAGGCCGTTTCGTTCCGTACAAAAGCAGTTTACAACCCGAAGGCCTTCATCCTGCACGCGGCATGGCTGGATCAGGCTTTCGCCCATTGTCCAAAATTCCCCACTGCTGCCTCCCGTAGGAGTCTGGGCCGTGTCTCAGTCCCAGTGTGGCTGGTCGTCCTCTCAGACCAGCTACAGATCGAAGGCTTGGTGAGCCTTTACCTCACCAACTACCTAATCTGCCATCGGCCGCTCCATTCGCGCAAGGTCTTGCGATCCCCTGCTTTCATCCGTAGATCGTATGCGGTATTAGCACAGCTTTCGCTGCGTTATCCCCCACGATTGGGCACGTTCCGATGTATTACTCACCCGTTCGCCACTCGCCGCCAGGATTGCTCCCGCGCTGCCGTTCGACTGCA

> Terrabacter ginsengisoli SLT32

TGCAAGTCGAACGGTGATGACAGGGCTTGCCTTGTCTGATCAGTGGCGAACGGGTGAGTAACACGTGAGTAACCTGCCCCAGACTCTGGGATAACCCCGGGAAACCGGAGCTAATACCGGATATGACACCAACGGGCATGCGCATGGTGTGGAAAGTTTTTCGGTCTGGGATGGACTCGCGGCCTATCAGCTTGTTGGTGAGGTAGTGGCTCACCAAGGCGACGACGGGTAGCCGGCCTGAGAGGGCGACCGGCCACACTGGGACTGAGACACGGCCCAGACTCCTACGGGAGGCAGCAGTGGGGAATATTGCACAATGGGCGAAAGCCTGATGCAGCGACGCCGCGTGAGGGATGACGGCCTTCGGGTTGTAAACCTCTTTCAGCAGGGAAGAAGCGCAAGTGACGGTACCTGCAGAAGAAGCACCGGCTAACTACGTGCCAGCAGCCGCGGTAATACGTAGGGTGCGAGCGTTGTCCGGAATTATTGGGCGTAAAGAGCTTGTAGGTGGTTTGTCGCGTCTGCTGTGAAAATCCGAGGCTCAACCTCGGACTTGCAGTGGGTACGGGCAGACTAGAGTGTGGTAGGGGAGACTGGAATTCCTGGTGTAGCGGTGGAATGCGCAGATATCAGGAGGAACACCGATGGCGAAGGCAGGTCTCTGGGCCATTACTGACACTGAGAAGCGAAAGCATGGGGAGCGAACAGGATTAGATACCCTGGTAGTCCATGCCGTAAACGTTGGGAACTAGGTGTGGGTCTCATTCCACGAGATCCGTGCCGCAGCTAACGCATTAAGTTCCCCGCCTGGGGAGTACGGCCGCAAGGCTAAAACTCAAAGGAATTGACGGGGGCCCGCACAAGCGGCGGAGCATGCGGATTAATTCGATGCAACGCGAAGAACCTTACCAAGGCTTGACATACACCGGAATCACTCAGAGATGGGTGCGTCTTCGGACTGGTGTACAGGTGGTGCATGGTTGTCGTCAGCTCGTGTCGTGAGATGTTGGGTTAAGTCCCGCAACGAGCGCAACCCTCGTTCTATGTTGCCAGCACGTCATGGTGGGGACTCATAGGAGACTGCCGGGGTCAACTCGGAGGAAGGTGGGGATGACGTCAAATCATCATGCCCCTTATGTCTTGGGCTTCACGCATGCTACAATGGCCGGTACAAAGGGCTGCGATACCGCGAGGTGGAGCGAATCCCAAAAAACCGGTCTCAGTTCGGATTGGGGTCTGCAACTCGACCCCATGAAGTCGGAGTCGCTAGTAATCGCAGATCAGCAACGCTGCGGTGAATACGTTCCCGGGCCTTGTACACACCGCCCGTCAAGTCACGAAAGTCGGTAACACCCGAAGCCGGTGGCCCAACTGTTTACAGAGGGAGCCGTCGAA

> Mesorhizobium amorphae SLT33

CCGTGGTCGCCTGCCTCCTTGCGGTTAGCACAGCGCCTTCGGGTAAAACCAACTCCCATGGTGTGACGGGCGGTGTGTACAAGGCCCGGGAACGTATTCACCGCGGCATGCTGATCCGCGATTACTAGCGATTCCAACTTCATGCACTCGAGTTGCAGAGTGCAATCCGAACTGAGATGGCTTTTGGAGATTAGCTCGACCTCGCGGTCTCGCTGCCCACTGTCACCACCATTGTAGCACGTGTGTAGCCCAGCCCGTAAGGGCCATGAGGACTTGACGTCATCCCCACCTTCCTCTCGGCTTATCACCGGCAGTCCCCTTAGAGTGCCCAACTGAATGATGGCAACTAAGGGCGAGGGTTGCGCTCGTTGCGGGACTTAACCCAACATCTCACGACACGAGCTGACGACAGCCATGCAGCACCTGTCACCGGTCCAGCCGAACTGAAGGGATCCATCTCTGGAAACCGCGACCGGGATGTCAAGGGCTGGTAAGGTTCTGCGCGTTGCTTCGAATTAAACCACATGCTCCACCGCTTGTGCGGGCCCCCGTCAATTCCTTTGAGTTTTAATCTTGCGACCGTACTCCCCAGGCGGGAAGCTTAATGCGTTAGCTGCGCCACCGACAGGTAAACCTGCCAACGGCTAGCTTCCATCGTTTACAGCGTGGACTACCAGGGTATCTAATCCTGTTTGCTCCCCACGCTTTCGCACCTCAGCGTCAGTACCGGACCAGTGAGCCGCCTTCGCCACTGGTGTTCCTCCGAATATCTACGAATTTCACCTCTACACTCGGAATTCCACTCACCTCTTCCGGACTCGAGATTGCCAGTATTAAAGGCAGTTCCAGGGTTGAGCCCTGGGATTTCACCCCTAACTTAACAATCCGCCTACGTGCGCTTTACGCCCAGTAAATCCGAACAACGCTAGCCCCCTTCGTATTACCGCGGCTGCTGGCACGAAGTTAGCCGGGGCTTCTTCTACGGTTACCGTCATTATCTTCACCGTTGAAAGAGCTTTACAACCCTAGGGCCTTCATCACTCACGCGGCATGGCTGGATCAGGCTTTCGCCCATTGTCCAATATTCCCCACTGCTGCCTCCCGTAGGAGTCTGGGCCGTGTCTCAGTCCCAGTGTGGCTGATCATCCTCTCAGACCAGCTATGGATCGTCGCCTTGGTAGGCCATTACCCCACCAACTAGCTAATCCAACGCGGGCTCATCCATCACCGATAAATCTTTCTCCCGAAGGACGTATACGGTATTAGCTCCAGTTTCCCGGAGTTGTTCCGTAGTGATGGGTAGATTCCCACGCGTTACTCACCCGTCTGCCGCTCCCCTTGCGGGGCGCTCGACTTGCA

>Streptomyces zaomyceticus SLT34

TGCAAGTCGAACGATGAAGCCCTTCGGGGTGGATTAGTGGCGAACGGGTGAGTAACACGTGGGCAATCTGCCCTTCACTCTGGGACAAGCCCTGGAAACGGGGTCTAATACCGGATAACACCGGCTTCCGCATGTCGCCTGGTGGAAAGCTCCGGCGGTGAAGGATGAGCCCGCGGCCTATCAGCTTGTTGGTGGGGTAATGGCCTACCAAGGCGACGACGGGTAGCCGGCCTGAGAGGGCGACCGGCCACACTGGGACTGAGACACGGCCCAGACTCCTACGGGAGGCAGCAGTGGGGAATATTGCACAATGGGCGAAAGCCTGATGCAGCGACGCCGCGTGAGGGATGACGGCCTTCGGGTTGTAAACCTCTTTCAGCAGGGAAGAAGCGAGAGTGACGGTACCTGCAGAAGAAGCGCCGGCTAACTACGTGCCAGCAGCCGCGGTAATACGTAGGGCGCAAGCGTTGTCCGGAATTATTGGGCGTAAAGAGCTCGTAGGCGGCTTGTCACGTCGGGTGTGAAAGCCCGGGGCTTAACCCCGGGTCTGCATCCGATACGGGCAGGCTAGAGTGTGGTAGGGGAGATCGGAATTCCTGGTGTAGCGGTGAAATGCGCAGATATCAGGAGGAACACCGGTGGCGAAGGCGGATCTCTGGGCCATTACTGACGCTGAGGAGCGAAAGCGTGGGGAGCGAACAGGATTAGATACCCTGGTAGTCCACGCCGTAAACGTTGGGAACTAGGTGTTGGCGACATTCCACGTCGTCGGTGCCGCAGCTAACGCATTAAGTTCCCCGCCTGGGGAGTACGGCCGCAAGGCTAAAACTCAAAGGAATTGACGGGGGCCCGCACAAGCAGCGGAGCATGTGGCTTAATTCGACGCAACGCGAAGAACCTTACCAAGGCTTGACATATACCGGAAAGCATTAGAGATAGTGCCCCCCTTGTGGTCGGTATACAGGTGGTGCATGGCTGTCGTCAGCTCGTGTCGTGAGATGTTGGGTTAAGTCCCGCAACGAGCGCAACCCTTGTCCTGTGTTGCCAGCATGCCCTTCGGGGTGATGGGGACTCACAGGAGACCGCCGGGGTCAACTCGGAGGAAGGTGGGGACGACGTCAAGTCATCATGCCCCTTATGTCTTGGGCTGCACACGTGCTACAATGGCCGGTACAAAGAGCTGCGATGCCGCGAGGCGGAGCGAATCTCAAAAAGCCGGTCTCAGTTCGGATTGGGGTCTGCAACTCGACCCCATGAAGTCGGAGTTGCTAGTAATCGCAGATCAGCATTGCTGCGGTGAATACGTTCCCGGGCCTTGTACACACCGCCCGTCACGTCACGAAAGTCGGTAACACCCGAAGCCGGTGGCCCAACCCCTTGTGGGAGGGA

>Streptomyces spororaveus SLT35

TCCCTCCCTTACGGGTTGGGCCACCGGCTTCGGGTGTTACCGACTTTCGTGACGTGACGGGCGGTGTGTACAAGGCCCGGGAACGTATTCACCGCAGCAATGCTGATCTGCGATTACTAGCAACTCCAACTTCATGGGGTCGAGTTGCAGACCCCAATCCGAACTGAGACCGGCTTTTTGAGATTCGCTCCACCTCACGGTATCGCAGCTCATTGTACCGGCCATTGTAGCACGTGTGCAGCCCAAGACATAAGGGGCATGATGACTTGACGTCGTCCCCACCTTCCTCCGAGTTGACCCCGGCGGTCTCCTGTGAGTCCCCATCACCCCGAAGGGCATGCTGGCAACACAGGACAAGGGTTGCGCTCGTTGCGGGACTTAACCCAACATCTCACGACACGAGCTGACGACAGCCATGCACCACCTGTATACCGACCACAAGGGGGGCACTATCTCTAATGCTTTCCGGTATATGTCAAGCCTTGGTAAGGTTCTTCGCGTTGCGTCGAATTAAGCCACATGCTCCGCTGCTTGTGCGGGCCCCCGTCAATTCCTTTGAGTTTTAGCCTTGCGGCCGTACTCCCCAGGCGGGGAACTTAATGCGTTAGCTGCGGCACCGACGACGTGGAATGTCGCCAACACCTAGTTCCCAACGTTTACGGCGTGGACTACCAGGGTATCTAATCCTGTTCGCTCCCCACGCTTTCGCTCCTCAGCGTCAGTAATGGCCCAGAGATCCGCCTTCGCCACCGGTGTTCCTCCTGATATCTGCGCATTTCACCGCTACACCAGGAATTCCGATCTCCCCTACCACACTCTAGCTAGCCCGTATCGAATGCAGACCCGAGGTTAAGCCTCGGGCTTTCACATCCGACGTGACAAGCCGCCTACGAGCTCTTTACGCCCAATAATTCCGGACAACGCTTGCGCCCTACGTATTACCGCGGCTGCTGGCACGTAGTTAGCCGGCGCTTCTTCTGCAGGTACCGTCACTTTCGCTTCTTCCCTGCTGAAAGAGGTTTACAACCCGAAGGCCGTCATCCCTCACGCGGCGTCGCTGCATCAGGCTTTCGCCCATTGTGCAATATTCCCCACTGCTGCCTCCCGTAGGAGTCTGGGCCGTGTCTCAGTCCCAGTGTGGCCGGTCGCCCTCTCAGGCCGGCTACCCGTCGTCGCCTTGGTGGGCCATTACCCCACCAACAAGCTGATAGGCCGCGGGCTCATCCTTCACCGCCGGAGCTTTCAACCCCCGCCCATGCAGGCAGGAGTGGTATCCGGTATTAGACCCCGTTTCCAGGGCTTGTCCCAGAGTGAAGGGCAGATTGCCCACGTGTTACTCACCCGTTCGCCACTAATCCACCCCGAAGGGCTTCATCGTTCGACTGCA

>Agromyces italicus SLT36

TTCGACGGCTCCCTCCACAAGGGTTAGGCCACCGGCTTCGGGTGTTACCGACTTTCATGACTTGACGGGCGGTGTGTACAAGGCCCGGGAACGTATTCACCGCAGCGTTGCTGATCTGCGATTACTAGCGACTCCGACTTCATGAGGTCGAGTTGCAGACCTCAATCCGAACTGAGACCGGCTTTTTGGGATTCGCTCCGCCTTACGACATCGCAGCCCTTTGTACCGGCCATTGTAGCATGCGTGAAGCCCAAGACATAAGGGGCATGATGATTTGACGTCATCCCCACCTTCCTCCGAGTTGACCCCGGCAGTCTCACATGAGTTCCCACCATTACGTGCTGGCAACATGCGACGAGGGTTGCGCTCGTTGCGGGACTTAACCCAACATCTCACGACACGAGCTGACGACAACCATGCACCACCTGTTCACGAGTGTCCAAAGAGTTGACCATTTCTGGCCCGTTCTCGTGTATGTCAAGCCTTGGTAAGGTTCTTCGCGTTGCATCGAATTAATCCGCATGCTCCGCCGCTTGTGCGGGCCCCCGTCAATTCCTTTGAGTTTTAGCCTTGCGGCCGTACTCCCCAGGCGGGGCGCTTAATGCGTTAGCTACGACACGGAAACCGTGGAAAGGTCCCCACATCTAGCGCCCAACGTTTACGGCGTGGACTACCAGGGTATCTAATCCTGTTCGCTCCCCACGCTTTCGCTCCTCAGCGTCAGTTACGGCCCAGAGAACTGCCTTCGCCATCGGTGTTCCTCCTGATATCTGCGCATTCCACCGCTACACCAGGAATTCCATTCTCCCCTACCGCACTCAAGTCTGCCCGTACCCACTGCAGGCTAGAGGTTGAGCCTCTAGTTTTCACAGCAGACGCGACAAACCGCCTACGAGCTCTTTACGCCCAATAATTCCGGACAACGCTCGGACCCTACGTATTACCGCGGCTGCTGGCACGTAGTTAGCCGGTCCTTTTTCTGCAAGTACCGTCAAGGCCGAAGCCCCTTCTTCCTTACTAAAAGCGGTTTACAACCCGAAGGCCGTCATCCCGCACGCGGCGTTGCTGCATCAGGCTTGCGCCCATTGTGCAATATTCCCCACTGCTGCCTCCCGTAGGAGTCTGGGCCGTGTCTCAGTCCCAGTGTGGCCGGTCACCCTCTCAGGCCGGCTACCCGTCGACGCCTTGGTGAGCCATTACCTCACCAACAAGCTGATAGGCCGCGAGTCCATCCCAGACCGAAAAACTTTCCACCCCAGATCATGCGATCCGAGGTCCTATCCGGTATTAGACGTCGTTTCCAACGCTTATCCCAGAGTCCAGGGCAGGTTACTCACGTGTTACTCACCCGTTCGCCACTAATCCACCAGAGCAAGCTCCAGCTTCATCGTTCGACTTGCA

> Mitsuaria sp. SLT37

GGTATCGCCCCCCTTGCGGTTAGGCTAACTACTTCTGGCAGAACCCGCTCCCATGGTGTGACGGGCGGTGTGTACAAGACCCGGGAACGTATTCACCGCGGCAAGCTGATCCGCGATTACTAGCGATTCCGACTTCACGCAGTCGAGTTGCAGACTGCGATCCGGACTACGACCGGGTTTCTGGGATTAGCTCCCCCTCGCGGGTTGGCAGCCCTCTGTCCCGGCCATTGTATGACGTGTGTAGCCCTACCCATAAGGGCCATGATGACCTGACGTCATCCCCACCTTCCTCCGGTTTGTCACCGGCAGTCTCATTAGAGTGCCCTTTCGTAGCAACTAATGACAAGGGTTGCGCTCGTTGCGGGACTTAACCCAACATCTCACGACACGAGCTGACGACGGCCATGCAGCACCTGTGTCCAGGTTCTCTTTCGAGCACTCCCGCATCTCTGCAGGATTCCTAGCATGTCAAGGGTAGGTAAGGTTTTTCGCGTTGCATCGAATTAAACCACATCATCCACCGCTTGTGCGGGTCCCCGTCAATTCCTTTGAGTTTCAACCTTGCGGCCGTACTCCCCAGGCGGTCAACTTCACGCGTTAGCTACGTTACTGAGAAGAAACCCTCCCAACAACCAGTTGACATCGTTTAGGGCGTGGACTACCAGGGTATCTAATCCTGTTTGCTCCCCACGCTTTCGTGCATGAGCGTCAGTACAGGTCCAGGGGATTGCCTTCGCCATCGGTGTTCCTCCGCATATCTACGCATTTCACTGCTACACGCGGAATTCCATCCCCCTCTACCGTACTCTAGCCATGCAGTCACAAATGCAGTTCCCAGGTTGAGCCCGGGGATTTCACATCTGTCTTGCATAACCGCCTGCGCACGCTTTACGCCCAGTAATTCCGATTAACGCTTGCACCCTACGTATTACCGCGGCTGCTGGCACGTAGTTAGCCGGTGCTTATTCTTCAGGTACCGTCATGAACCCCCAGTATTAGTAGGAGTCTTTTCTTCCCTGACAAAAGCGGTTTACAACCCGAAGGCCTTCTTCCCGCACGCGGCATGGCTGGATCAGGGTTGCCCCCATTGTCCAAAATTCCCCACTGCTGCCTCCCGTAGGAGTCTGGGCCGTGTCTCAGTCCCAGTGTGGCTGGTCGTCCTCTCAGACCAGCTACAGATCGTAGGCTTGGTAGGCCTTTACCCCACCAACTACCTAATCTGATATCGGCCGCTCCAATTGCGCGAGGTCTTGCGATCCCCCGCTTTCACCCTCAGGTCGTATGCGGTATTAGCTGCTCTTTCGAGCAGTTATCCCCCACAACTGGGCACGTTCCGATATATTACTCACCCGTTCGCCACTCGTCGCCAGGTGCCCCGCGTTACCGTTCGACTT

>Staphylococcus epidermidis SLT38

CAAGTCGAGCGAACAGACGAGGAGCTTGCTCCTCTGACGTTAGCGGCGGACGGGTGAGTAACACGTGGATAACCTACCTATAAGACTGGGATAACTTCGGGAAACCGGAGCTAATACCGGATAATATATTGAACCGCATGGTTCAATAGTGAAAGACGGTTTTGCTGTCACTTATAGATGGATCCGCGCCGCATTAGCTAGTTGGTAAGGTAACGGCTTACCAAGGCAACGATGCGTAGCCGACCTGAGAGGGTGATCGGCCACACTGGAACTGAGACACGGTCCAGACTCCTACGGGAGGCAGCAGTAGGGAATCTTCCGCAATGGGCGAAAGCCTGACGGAGCAACGCCGCGTGAGTGATGAAGGTCTTCGGATCGTAAAACTCTGTTATTAGGGAAGAACAAATGTGTAAGTAACTATGCACGTCTTGACGGTACCTAATCAGAAAGCCACGGCTAACTACGTGCCAGCAGCCGCGGTAATACGTAGGTGGCAAGCGTTATCCGGAATTATTGGGCGTAAAGCGCGCGTAGGCGGTTTTTTAAGTCTGATGTGAAAGCCCACGGCTCAACCGTGGAGGGTCATTGGAAACTGGAAAACTTGAGTGCAGAAGAGGAAAGTGGAATTCCATGTGTAGCGGTGAAATGCGCAGAGATATGGAGGAACACCAGTGGCGAAGGCGACTTTCTGGTCTGTAACTGACGCTGATGTGCGAAAGCGTGGGGATCAAACAGGATTAGATACCCTGGTAGTCCACGCCGTAAACGATGAGTGCTAAGTGTTAGGGGGTTTCCGCCCCTTAGTGCTGCAGCTAACGCATTAAGCACTCCGCCTGGGGAGTACGACCGCAAGGTTGAAACTCAAAGGAATTGACGGGGACCCGCACAAGCGGTGGAGCATGTGGTTTAATTCGAAGCAACGCGAAGAACCTTACCAAATCTTGACATCCTCTGACCCCTCTAGAGATAGAGTTTTCCCCTTCGGGGGACAGAGTGACAGGTGGTGCATGGTTGTCGTCAGCTCGTGTCGTGAGATGTTGGGTTAAGTCCCGCAACGAGCGCAACCCTTAAGCTTAGTTGCCATCATTAAGTTGGGCACTCTAAGTTGACTGCCGGTGACAAACCGGAGGAAGGTGGGGATGACGTCAAATCATCATGCCCCTTATGATTTGGGCTACACACGTGCTACAATGGACAATACAAAGGGTAGCGAAACCGCGAGGTCAAGCAAATCCCATAAAGTTGTTCTCAGTTCGGATTGTAGTCTGCAACTCGACTATATGAAGCTGGAATCGCTAGTAATCGTAGATCAGCATGCTACGGTGAATACGTTCCCGGGTCTTGTACACACCGCCCGTCACACCACGAGAGTTTGTAACACCCGAAGCCGGTGGAGTAACCATTTGGAGCTAGCCGTCGAA

> Bacillus velezensis LT1 *gyrA*

GGAATGCATGAGCGTTATCGTATCCCGGGCGCTTCCGGATGTGCGTGACGGTCTGAAGCCGGTTCACAGACGGATTTTGTACGCAATGAATGATTTAGGCATGACCAGTGACAAACCATATAAAAAATCTGCCCGTATCGTCGGTGAAGTTATCGGTAAGTACCACCCGCACGGTGACTCAGCGGTTTACGAATCAATGGTCAGAATGGCGCAGGATTTTAACTACCGCTACATGCTTGTTGACGGACACGGCAACTTCGGTTCGGTTGACGGCGACTCAGCGGCCGCGATGCGTTACACAGAAGCGAGAATGTCAAAAATCGCAATGGAAATTCTGCGTGACATTACGAAAGACACGATTGACTATCAAGATAACTATGACGGTTCAGAAAGAGAGCCTGCCGTCATGCCTTCGAGATTTCCGAATCTGCTCGTAAACGGGGCTGCCGGTATTGCGGTCGGAATGGCGACAAACATTCCCCCGCATCAGCTTGGGGAAGTCATTGAAGGCGTGCTTGCCGTAAGTGAGAATCCTGAGATTACAAACCAGGAGCTGATGGAATACATCCCGGGCCCGGATTTTCCGACTGCAGGTCAGATTTTGGGCCGGAGCGGCATCCGCAAGGCATATGAATCCGGACGGGGATCAATCACGATCCGGGCTAAGGCTGAAATCGAAGAGACTTCATCGGGAAAAGAAAGAATTATTGTCACGGAACTTCCTTATCAGGTGAACAAAGCGAGATTAATTGAAAAAATCGCGGATCTTGTCCGGGACAAAAAAATCGAAGGAATTACCGATCTGCGAGACGAATCCGACCGTAACGGAATGAGAATCGTCATTGAGATCCGCCGTGACGCCAATGCTCACGTCATTTTGAATAACCTGTACAAACAAACGGCCCTGCAGACGTCTTTCGGAATCAACCTGCTGGCGCTCGTGACGGACAGCCGAAAAGT
