# Supplementary material for: Bacillus velezensis LT1: a potential biocontrol agent for southern blight on Coptis chinensis
Source: Front Microbiol. 2024 Mar 4;15:1337655. doi: 10.3389/fmicb.2024.1337655 (PMC10946422; doi:10.3389/fmicb.2024.1337655)
Supplement: Supplementary file 2 [file Data_Sheet_2.docx]

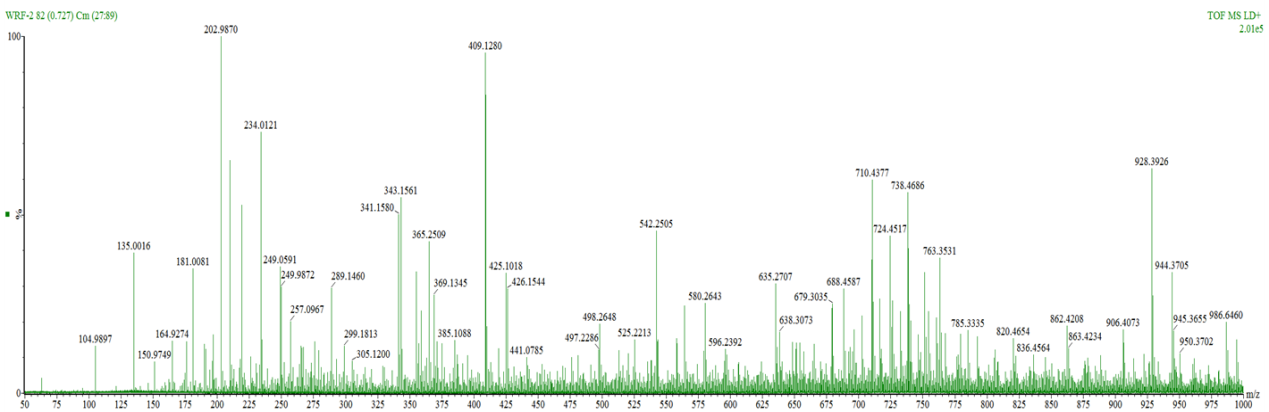


MALDI-TOF-MS spectrum at m/z 50～1000 of lipopeptide components of *Bacillus velezensis* LT1

MALDI-TOF-MS spectrum at m/z 1000～2000 of lipopeptide components of *Bacillus velezensis* LT1

MALDI-TOF-MS spectrum at m/z 1000～1100 of lipopeptide components of *Bacillus velezensis* LT1

MALDI-TOF-MS spectrum at m/z 1100～1200 of lipopeptide components of *Bacillus velezensis* LT1

MALDI-TOF-MS spectrum at m/z 1200～1300 of lipopeptide components of *Bacillus velezensis* LT1

MALDI-TOF-MS spectrum at m/z 1300～1400 of lipopeptide components of *Bacillus velezensis* LT1

MALDI-TOF-MS spectrum at m/z 1400～1500 of lipopeptide components of *Bacillus velezensis* LT1

MALDI-TOF-MS spectrum at m/z 1500～1600 of lipopeptide components of *Bacillus velezensis* LT1
